# A covalent BTK ternary complex compatible with targeted protein degradation

**DOI:** 10.1038/s41467-023-36738-z

**Published:** 2023-03-02

**Authors:** James Schiemer, Andrew Maxwell, Reto Horst, Shenping Liu, Daniel P. Uccello, Kris Borzilleri, Nisha Rajamohan, Matthew F. Brown, Matthew F. Calabrese

**Affiliations:** Discovery Sciences, Pfizer Worldwide Research and Development, Groton, CT USA

**Keywords:** X-ray crystallography, Screening, Ubiquitylation, Structure-based drug design

## Abstract

Targeted protein degradation using heterobifunctional chimeras holds the potential to expand target space and grow the druggable proteome. Most acutely, this provides an opportunity to target proteins that lack enzymatic activity or have otherwise proven intractable to small molecule inhibition. Limiting this potential, however, is the remaining need to develop a ligand for the target of interest. While a number of challenging proteins have been successfully targeted by covalent ligands, unless this modification affects form or function, it may lack the ability to drive a biological response. Bridging covalent ligand discovery with chimeric degrader design has emerged as a potential mechanism to advance both fields. In this work, we employ a set of biochemical and cellular tools to deconvolute the role of covalent modification in targeted protein degradation using Bruton’s tyrosine kinase. Our results reveal that covalent target modification is fundamentally compatible with the protein degrader mechanism of action.

## Abstract (web summary)

Bridging covalent ligand discovery with chimeric degrader design has emerged as a mechanism to target proteins that lack enzymatic activity or are intractable. Here, the authors use biochemical and cellular tools to deconvolute the role of covalent modification in targeted protein degradation using Bruton’s tyrosine kinase.

## Introduction

Over the past two decades, the application of chimeric degraders has rapidly expanded from chemical biology tools to clinical assets^1,2^. Despite tremendous progress, the majority of advanced chimeric degraders still focus on well-validated ligandable targets. This underscores a critical gap in the field, as we witness a growing list of genetically validated proteins that are devoid of discernable small molecule binding sites^3^. One approach to address this challenge lies in the development of covalent ligands, which often utilize an electrophilic functional group to target a cryptic pocket and partially compensate for limited non-covalent binding affinity^4^. While this presents an opportunity to ligand even non-enzymatic targets, it may still present a gap in terms of modulating a pharmacodynamic response^4–7^. This has led to growing interest in incorporating these ligands as components of protein degraders^8–10^.

Covalency in chimeric degraders has been widely explored in the context of E3 ligase binders^11–13^. This strategy has the advantage of expanding the E3 ligase toolbox for TPD, while preserving the catalytic nature of chimeric degraders^14^, as a covalent E3-ligand complex can still take part in multiple rounds of substrate turnover. In contrast, covalent modification on the target protein may reduce the catalytic activity of this mechanism, which may raise the dose required to achieve a therapeutic level of degradation, depending on the protein levels and the rate of resynthesis. Nevertheless, chimeric degraders that covalently modify targets have been conceptually validated from the first degrader reported by Sakatomoto et al targeting MetAP-2^1^, to more recent examples involving KRAS and ERK1/2^15–19^. However, while these studies are supportive, they do not demonstrate that a stable covalent modification actually precedes cellular degradation. Similarly, while approaches such as HaloTag-targeting chloroalkane degraders (HaloPROTACs)^20,21^ provide valuable chemical biology tools to interrogate target function, they provide only limited insights into covalent degrader function, owing to the more labile nature of the ester bonds utilized.

Bruton’s Tyrosine Kinase (BTK) represents a therapeutically relevant protein that can be explored for covalent degraders. BTK, a validated target for B-cell malignancies, presents a reactive cysteine (C481) in the ATP binding pocket of the kinase domain that has been co-opted for the development of potent covalent inhibitors, including approved therapeutics such as ibrutinib^22,23^. The rise of clinical mutations that are refractory to current therapies has presented a need for new therapeutic approaches, including degraders. Prior work has established that BTK is a robust target for non-covalent and reversible covalent chimeric degraders, with several molecules progressing to Phase 1 clinical trials (Beigene (NCT05006716); Nurix (NCT04830137, NCT05131022); Haisco (NCT04861779)). However, differing reports as to whether covalent irreversible BTK chimeras can drive efficient degradation have resulted in uncertainty around the consequences and context of the covalent modification itself^24–28^.

Finally, while the protein degrader mechanism of action is predicated on the formation of a productive target-degrader-E3-ligase ternary complex, structural insights into these complexes remain few, with no examples yet representing a covalent assembly^29–39^. In addition, despite its wide utility in the protein degrader field, the cellular inhibitor of apoptosis (cIAP1) remains limited in terms of deep mechanistic characterization^40^.

We, therefore, set out to expand our understanding of target recruitment within the framework of covalent degraders targeting BTK and co-opting cIAP1. Here, we show that covalently modified BTK is a bonafide target for proteasomal degradation and structurally represents yet another unique ternary complex pose for this protein combination.

## Results

### BCCov degrades BTK

**Fig. 1:**
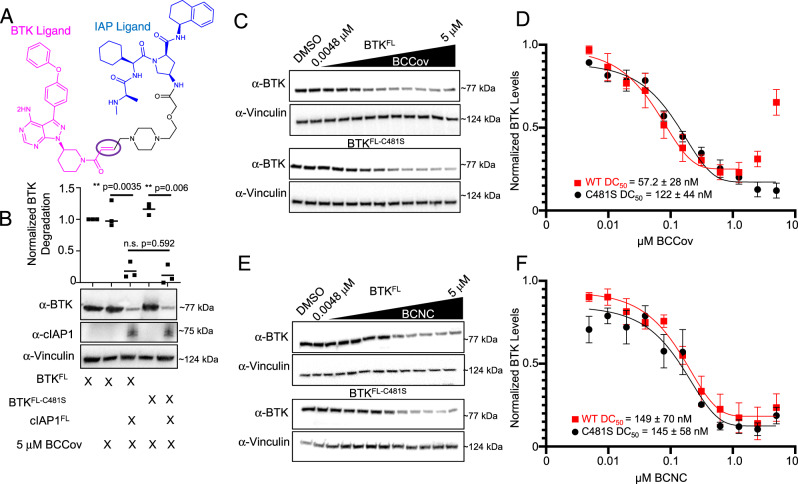
Degradation of BTK via covalent and non-covalent chimeric degraders. **A** Schematic representation of BCCov. Regions corresponding to the BTK warhead (magenta), linker (black), and IAP warhead (blue) are indicated. Reactive alkene circled in purple. **B** Western blot of transiently transfected BTK^FL^, BTK^FL-C481S^, and/or cIAP1^FL^ with or without 5 µM BCCov treatment. Degradation of BTK across 3 separate experiments were quantified and normalized to untreated BTK^FL^ and plotted as a scatter plot around the median. Representative western blots for BCCov (**C**, **D**) and BCNC (**E**, **F**) mediated dose-dependent degradation of BTK^FL^ and BTK^FL-C481S^. Quantification and DC_50_ plot for degradation of BTK^FL^ or BTK^FL-C481S^ across 3 separate experiments shown in panel. Mean values were plotted with SEM Error bars. Significance was determined using a two-sided unpaired student’s T-Test, where significance is denoted with a *p*-value ≤0.05 (*), and ≤0.01 (**). Samples processed from different blots were from the same experiment and processed in parallel using DMSO treatment as a normalization control.

As a starting point for our work, we selected an irreversible covalent degrader (referred to throughout this study as BTK-cIAP1-Covalent degrader (BCCov)), from a previously disclosed series built from an Ibrutinib-like BTK warhead fused to a known ligand of the IAP family of E3 ligases^26,41^ (Fig. 1A). To provide a comparator compound incapable of forming a covalent bond with BTK, an analog of BCCov was prepared where the reactive acrylamide double bond was replaced with a non-reactive single bond, referred to hereafter as BTK-cIAP1-Non-Covalent degrader (BCNC) as reported previously (Tinworth et al. Compounds 6, 7)^26,41^.

Prior studies have shown differing results for covalent irreversible BTK degraders with some reports of potent and robust activity^24,27^, while others show only weak or ineffective degradation^25,26^. However, results are sometimes complicated by the fact that many of these degraders still retain a high affinity for BTK, even in the absence of covalent modification. In order to delineate covalent modification from degradation, we established an adaptable system using HEK293 cells (Expi293). In contrast to the B-cell lineage, Expi293 cells lack endogenous expression of BTK. Combined with a low apparent basal expression of cIAP1, they provide an opportunity to exogenously regulate cellular degradation conditions. Thus, using a transient transfection system to introduce full-length BTK (BTK^FL^), BTK^FL-C481S^_,_ and full-length cIAP1 (cIAP1^FL^) (Supplementary Fig. 1), we generated a system that is responsive to cIAP1-dependent BCCov-mediated degradation of BTK^FL^ (Fig. 1B).

To examine the importance of covalent modification we performed dose-dependent degradation using BCNC and BCCov with cells expressing either BTK^FL^ or BTK^FL-C481S^. Consistent with previous studies^24,42^, we observe similar DC_50_ values throughout (Fig. 1C–F) suggesting an ability of BCCov to function through both covalent and non-covalent recruitment of BTK. In order to validate degradation specificity, we generated a BTK gatekeeper mutant (BTK^T474I^) as well as a C481S/T474I double mutant (BTK^T474I-C481S^) in which the ligand binding ability of BTK is impaired (Supplementary Fig. 2A)^43^ and observed the expected loss of activity. Finally, in order to compare to a more relevant endogenous system, we used a THP-1 cell line which natively expresses both BTK and cIAP1 and obtain DC_50_ values that are in-line with our transient transfection system (Supplementary Fig. 2B, C).

### BCCov can covalently modify BTK in cells

**Fig. 2:**
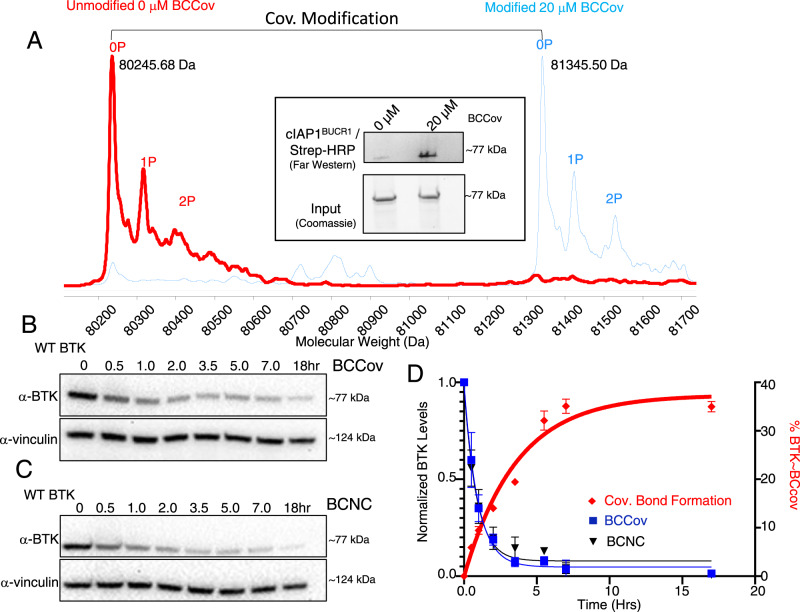
BCCov covalently labels BTK in cells. **A** Deconvoluted intact mass spectrum chromatograph of immunopurified BTK^FL^ after DMSO treatment (red) or after cellular treatment with 20 µM BCCov (blue). Caliper indicates mass shift representing BCCov modification. Heterogeneous phosphorylation states 0P, 1P, or 2P represent 79.9 Da mass shifts. The inlay shows a Coomassie-stained gel showing Immunopurified BTK^FL^ and corresponding far western blot with biotinylated-cIAP1^BUCR1^ detected with streptavidin HRP. Each is a representative image for 3 independent experiments. **B**, **C** Representative western blot for time-dependent degradation of BTK^FL^ and vinculin loading controls after 5 µM BCCov or BCNC treatment, respectively. Each experiment was performed 3 times. **D** Graph of % covalent bond formation (red diamonds, right axis) versus observed BTK^FL^ degradation (blue squares and black triangles for BCCov and BCNC respectively; left axis) over time. Each experiment was performed three times, with mean values plotted with SEM Error bars. Samples processed from different blots were from the same experiment and processed in parallel using DMSO treatment as a normalization control.

Although covalent modification of BTK does not appear to be required for degradation by BCCov (Fig. 1C–F) we wished to explore whether covalent modification can and does occur within the cellular context. As a first step, we transfected Expi293 cells with BTK^FL^ but without cIAP1^FL^, a condition that promoted no observable ligand-induced BTK degradation (Fig. 1B, lane 2). Using a high concentration of BCCov (20 µM) with a single overnight time point (18 h), we performed denaturing intact mass spectrometry on immunopurified BTK^FL^ and observed a mass-shift consistent with the [BTK~BCCov] covalent adduct for nearly the entire detected population of BTK^FL^ (Fig. 2A, Supplementary Table 1). In order to confirm the assignment of this modification (~BCCov), we performed a far western blot on this immunopurified BTK^FL^ using recombinant biotinylated cIAP1^BUCR1^ which is capable of binding to the IAP warhead exposed in the [BTK~BCCov] complex (Fig. 2A, inlay). Using Streptavidin Horse Radish Peroxidase (HRP) to detect the biotinylated cIAP1^BUCR1^, we obtain results mirroring the mass spectrometry profile and validating the identity of the BTK adduct as [BTK~BCCov]. A parallel experiment using BTK^FL-C481S^ reveals no similar adduct, confirming the specificity of the modification (Supplementary Fig. 2D). Taken together, these data suggest that covalent [BTK~BCCov] formation can occur in this context.

In order to test whether covalent modification precedes BTK degradation, we performed a set of parallel time-course studies (Fig. 2B–D; Supplementary Fig. 3A, B). In one assay BTK^FL^ was co-transfected with cIAP1^FL^, treated with 5 µM BCCov and degradation was measured by western blot over time. In the second assay, BTK^FL^ was transfected in the absence of exogenous cIAP1, treated with an equivalent amount of BCCov, and analyzed by mass spectrometry at matched intervals. As shown in Fig. 2B–D, we observed degradation starting as early as 30 min and reaching a plateau by the 7-hour timepoint. Intriguingly, covalent modification occurs on a similar, though slightly slower time scale, suggesting that covalently modified BTK may form during the degradation time course. By comparison, BTK^FL-C481S^ degrades at a slower rate, likely reflecting a slightly reduced affinity of the BTK warhead toward this mutant^42,44^ (Supplementary Fig. 3A, B). Thus, although timescales appear compatible, the ability of modified BTK to serve as a target for the proteasome remains unclear.

### Covalently-labelled BTK can be degraded

**Fig. 3:**
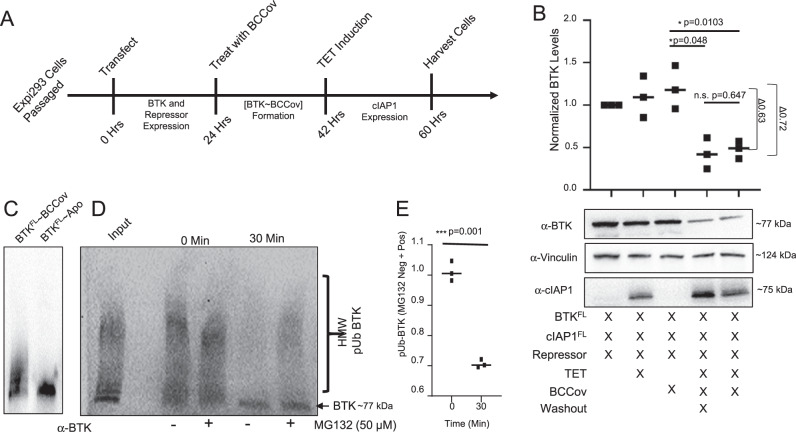
Covalently modified BTK is degraded. **A** Summary of stepwise protocol to pre-form a binary BTK^FL^~BCCov complex and induce cIAP1^FL^ using tetracycline. **B** Western blot analysis of BTK^FL^, vinculin, and cIAP1^FL^ with or without BCCov treatment, tetracycline induction, and compound washout. Mean values from 3 individual experiments were normalized to lane 1 and plotted as a scatter plot around the median. **C** Western blot of BCCov-dependent in vitro ubiquitination of BTK^FL^. The western is a representative blot from 3 independent experiments. **D** Western blot depicting in vitro (lysate) proteasomal degradation of ubiquitinated BTK^FL^~BCCov. Treatment of 50 µM proteasomal inhibitor MG132 indicated below. **E** Densiometric quantification of high molecular weight (HMW) ubiquitinated BTK^FL^ species comparing MG132 negative samples to MG132 positive samples. Values from **D**, and **E** are averaged over three independent experiments and plotted as a scatter plot around the median. Significance was determined using a two-sided unpaired student’s T-Test, significance is denoted with a *p*-value ≤0.05 (*), ≤0.01 (**), and ≤0.001 (***).

To more convincingly demonstrate whether covalently modified BTK is capable of serving as a degradation substrate, we used our transient transfection system to conduct sequential modification and degradation assays. We first assessed the fraction of cellular BTK that could be modified after an 18-hour incubation, with increasing concentrations of BCCov in the absence of transfected cIAP1 (Supplementary Fig. 3C). Using immunoprecipitation and mass spectrometry, we observe that after treatment with a 20 µM dose of BCCov we can reproducibly achieve approximately 94% BTK covalent modification (*n* = 5, 93.6 ± 1.7%), and this level remains stable for at least 48 h post-treatment (Supplementary Fig. 3D). Importantly, we did not observe a non-specific BTK^FL^~BCCov population forming as a result of the immunoprecipitation process itself (Supplementary Fig. 3E). In order to test for degradation of pre-formed BTK~BCCov covalent complex, we established an inducible (T-REx) cIAP1^FL^ system, in which expression of cIAP1^FL^ is turned on only upon cell treatment with tetracycline (Supplementary Fig. 3F). In the absence of tetracycline, our pool of [BTK^FL^~BCCov] remains intact, consistent with repression of cIAP1^FL^ expression. However, induction of cIAP1^FL^ promotes robust degradation of BTK^FL^ with D_max_ ~70%, demonstrating strong reduction of the starting [BTK^FL^~BCCov] pool (Fig. 3A, B). In order minimize possible contributions from engagement of any newly synthesized BTK, we included an additional experiment with extensive compound washout after BTK labeling which yielded consistent results.

As an additional orthogonal study, we performed an in vitro degradation assay using covalently modified recombinant BTK. In brief, purified BTK^FL^ was covalently labelled with BCCov and repurified (desalted) to remove excess compound (Supplementary Fig. 3G). This [BTK^FL^~BCCov] was then subjected to in vitro ubiquitination, generating a distribution of high molecular weight ubiquitinated conjugates (Fig. 3C). Addition of this sample to our Expi293 lysate shows loss of ubiquitinated [BTK^FL^~BCCov] in a proteasome-dependent manner, confirming compatibility of this degradation substrate (Fig. 3D, E).

### [BTK~BCCov] can form a productive ternary complex

Based on the cellular results above, we moved on to characterize the interactions between BTK, BCCov, and cIAP1. This was accomplished using an in vitro reconstituted system comprised of BTK’s kinase domain (BTK^KD^) and an enzymatically active truncated form of cIAP1 (cIAP1^BUCR1^)^45,46^ as previously described^40^ (Supplementary Fig. 1). To remove complexities arising from kinetics of covalent bond formation, we started with a pre-formed a BTK^KD^~BCCov irreversible binary complex, [BTK^KD^~BCCov]. As before, complex formation was measured by intact mass spectrometry with desalting to remove excess degrader (Supplementary Fig. 4A; Supplementary Table 1).

Our first assay utilized size exclusion chromatography coupled to multi-angle light scattering (SEC MALS) to assess E3 ligase recruitment (Supplementary Fig. 4B)^47,48^. In this experiment, while the interaction between the covalent BTK~BCCov complex and cIAP1 is formally described as a binary interaction, we maintain the ternary complex nomenclature in order to align with established norms in the protein degradation field in reference to target-chimera-E3 ligase complexes. Though some conformational heterogeneity is apparent in the major peak, analysis reveals the formation of a complex between BTK^KD^~BCCov and cIAP1^BUCR1^, as well as a [cIAP1^BUCR1^-BCNC] and BTK^KD^ complex, with an approximate 2:2 or 1:2 stoichiometry (BTK^KD^: cIAP1^BUCR1^), consistent with our prior report with non-covalent analogs^40^, and aligned with chemical cross-linking tracked by SDS-PAGE (Supplementary Fig. 4C, D). Lastly, we observe a cIAP1-dependent ubiquitination of BTK^KD^~BCCov, suggesting no fundamental defect in ternary complex formation and ubiquitin transfer (Supplementary Fig. 4E).

Next, we characterized the kinetics and energetics behind the formation of the covalent ternary complex. Here we used biolayer interferometry (BLI), to analyze BTK^KD^~BCCov binding to cIAP1^BUCR1^ as a measurement of complex formation. We demonstrated a relatively tight ternary complex (K_d_ = 72.5 nM), similar to a [cIAP1^BUCR1^ -BCNC] binary complex binding BTK^KD^ (49.8 nM), suggesting that the cIAP1 affinity is not substantially impacted by the BTK~BCCov covalent bond (Supplementary Fig. 5A, B). Interestingly, while the ternary complex affinity remains relatively high, it is nevertheless weaker than the binary affinity measured against cIAP1 alone (Supplementary Fig. 5C). While the tight affinity and slow off-rate of BCCov to cIAP1^BUCR1^ presented a challenge in determination of absolute K_d_ we can confidently assign an affinity of ≤ 10 nM, which is in close agreement with the ~16 nM affinity reported for the IAP warhead alone^41^. This allows us to set an upper limit for the cooperativity factor (α = K_d_ binary/K_d_ ternary) of ≤0.2 for BCCov, suggesting a relatively tight, yet negatively cooperative ternary complex.

### BCCov drives a distinct ternary complex structure

**Fig. 4:**
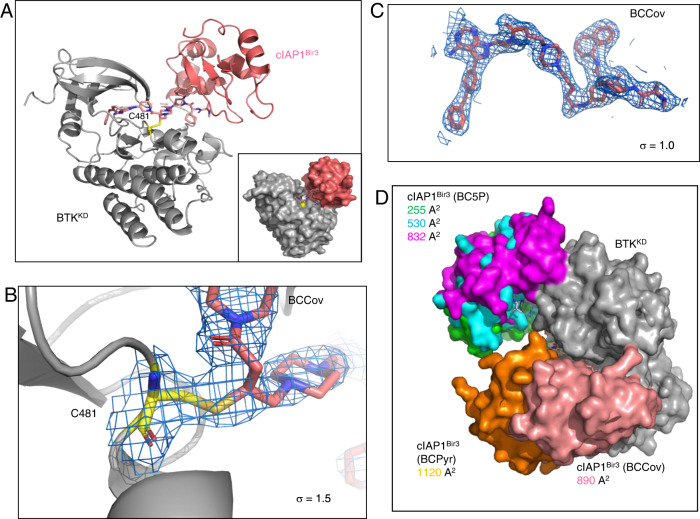
Ternary complex crystal structure of BTK^KD^~BCCov-cIAP1^Bir3^. **A** Cartoon depiction of BTK^KD^ (gray), BCCov (elemental/pink), cIAP1^Bir3^ (salmon), cysteine 481 backbone highlighted in yellow, and a surface model (inlay). **B** Cysteine 481 (yellow) shows continuous electron density to BCCov at stringent 2Fo-Fc density (σ = 1.5). **C** Electron density at 2Fo-Fc density (σ = 1.0) surrounding BCCov. **D** Surface model of BTK^KD^ (gray) and cIAP1^Bir3^ in unique crystallographic poses driven by 3 separate degraders; BC5P (magenta, light blue, green; 6W8I), BCPyr (orange; 6W7O), and BCCov (salmon) with a respective buried surface area at the ternary complex interface.

In order to gain deeper insights into the topology and interactions of the BTK covalent ternary complex, we shifted our efforts to structural studies. Focusing on the ligand binding domains of both BTK (kinase domain – BTK^KD^) and cIAP1 (Bir3 domain–cIAP1^Bir3^), we performed extensive crystallization trials in the presence of stoichiometric BCCov - ultimately yielding a 3.0 Å (2.3 Å anisotropic) structure of the ternary complex in which the covalent attachment of BCCov to Cysteine 481 of BTK is unambiguously resolved (Fig. 4A, B; Supplementary Table 2). The resulting electron density map provides clear density over the entirety of the ligand, providing high confidence in modeled trajectory and geometry, and reveals yet another ternary complex pose for this target/E3 ligase pair^40^ (Fig. 4C, D).

**Fig. 5:**
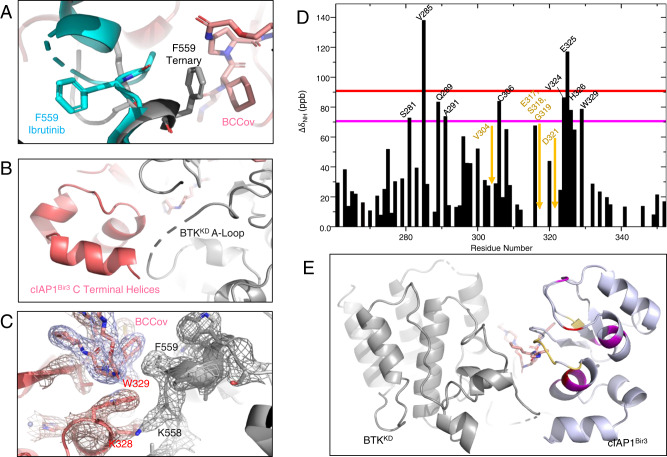
Detailed ternary complex interactions. **A** Hydrophobic interaction between BTK residue F559 (gray) and the cyclohexyl moiety (elemental/pink) on the cIAP1 warhead, and the conformational change it undergoes as compared to the deposited structure of a BTK^KD^-ibrutinib complex (blue, RCSB 5P9J). **B** C-Terminal helices of cIAP1^Bir3^ clash with the unstructured A loop of BTK^KD^. **C** Close lipophilic interactions at the ternary complex interface. Electron density mesh resolved at σ = 1.0. **D** Chemical shift variations of cIAP1^Bir3^–BCCov upon binding of BTK^KD^. Δδ_NH_ is the weighted average of the ^15^N and ^1^H chemical shifts^40^ with Δδ_NH_ values above 70, and 90 ppb demarcated as magenta and red respectively. cIAP1^Bir3^ signals that are broadened beyond detection in presence of BTK^KD^ are highlighted in orange, and those that experience large chemical shift perturbations are highlighted in black. **E** Ternary complex of BTK^KD^ (gray), cIAP1^Bir3^ (light blue), and BCCov (elemental/pink) with cIAP1^Bir3^ additionally colored at sites of chemical shift perturbations specific to the ternary complex as in **D**.

While modest electron density precludes complete side-chain modeling, we can nevertheless observe conformational changes in BTK that are different from the previously reported ibrutinib-bound structure (RCSB 5P9J^49^). This includes a striking outward flip of F559 allowing this aromatic sidechain to pack against the cyclohexyl moiety of the cIAP1 ligand of BCCov within the context of the ternary complex, highlighting the flexibility and induced contacts in this non-native assembly (Fig. 5A). Interestingly, the cyclohexyl group of BCCov ligand also appears dynamic and adopts a unique rotation as compared to the binary BCCov-cIAP1^Bir3^ structure that we have additionally solved for comparison (Supplementary Fig. 5D, E). Nevertheless, despite the apparent favorable interaction between F559 and BCCov, this contact appears to be dispensable for BCCov-mediated BTK degradation, as the F559A mutant retains potent cellular degradation, binding kinetics, and ternary complex formation (Supplementary Fig. 6), suggesting that there is sufficient plasticity in the accessible conformations to compensate energetically. Finally, we observe a series of intimate lipophilic contacts between BTK^KD^ and cIAP1^Bir3^ including several proximal to the F559/BCCov interaction with alternating residues zippering at the interface (Fig. 5C).

### Ternary complex solution ensemble

To validate our crystallographic findings and assess the conformation of BTK^KD^-BCCov-cIAP1^Bir3^ in solution, we performed 2-dimensional (2D) NMR experiments as previously described^40^. Here, uniformly [^13^C,^15^N]-labelled cIAP1^Bir3^ was pre-incubated with either BCCov [cIAP1^Bir3^ + BCCov] to generate a cIAP1^Bir3^-BCCov binary complex, or ([cIAP1^Bir3^-BCCov] + BTK^KD^) to generate a ternary complex (Supplementary Fig. 7A). Addition of BTK leads to perturbations in residues within the C-terminal helices and preceding loops (Fig. 5D, E), consistent with the interface observed in the crystal structure (Fig. 5B, C). In addition, severe line-broadening occurred for a specific set of residues near the cIAP1 binding site (Fig. 5D) which is indicative of slow conformational exchange on the μs-to-ms time scale and may reflect multiple binding modes of BCCov to cIAP1^Bir3,^^50^.

## Discussion

The emergence of covalent reversible and irreversible protein degraders potentiates an exciting new avenue to modulate degradation extent and specificity. While there is a theoretical predicted loss of catalytic degradation, irreversible covalent degraders may provide an attractive approach against at least a subset of targets^24,27^. Here, we present a study of an irreversible-covalent degrader (BCCov) of BTK as the target, and cIAP1 as the E3 ligase. We demonstrate that a covalent bond can be formed prior to proteolysis, illustrating that covalent ligands are fundamentally compatible with targeted protein degradation.

We first determined that BCCov leads to potent degradation of BTK. In order to dissect this process, we established an engineered cellular system that allowed us to interrogate the distinct steps in this process through selective expression of both target and E3 ligase. By limiting the expression of cIAP1, we confirm the ability of BCCov to covalently modify BTK in cells, in a timeframe that is consistent with the observed kinetics of degradation.

To confirm that covalently modified BTK is compatible with proteasomal degradation, we established a tetracycline-inducible cellular system to allow us to pre-form a stable population of covalently modified BTK~BCCov, and subsequently initiate the degradation of this labelled pool. In parallel, we generated ubiquitinated BTK~BCCov which we utilized as a substrate for in vitro degradation assays in cell lysate. Here we note that prior reports in different cellular systems have shown much weaker or limited BTK degradation with analogous covalent molecules to those reported here^25,26^. However, the context-dependent nature of degrader potency across cell types and systems has been broadly noted and may relate to alterations in abundance and functional state of target and E3 ligase, alterations in protein resynthesis rates, as well as changes in post-translational modifications or recruitment to binding partners and complexes – any or all of which may impact degrader potency. We, therefore, caution that such model systems are used most appropriately to demonstrate mechanistic compatibility, rather than to interrogate absolute potency.

After establishing that BTK~BCCov is a competent species for degradation, we turned to a suite of in vitro assays to characterize the biophysics and structure of its recruitment to the E3 ligase cIAP1. Using SPR, NMR, multi-angle light scattering, and chemical crosslinking, we unambiguously confirm the ability of this BTK~BCCov to take part in a stable ternary complex with cIAP1. While this complex lacks positive thermodynamic cooperativity (*α* ≤ 0.2), it is nevertheless capable of promoting cIAP1-mediated ubiquitination, demonstrating productive recruitment to the E3 ligase machinery.

Turning to x-ray crystallography, coupled with NMR, we observe a stable yet dynamic ternary complex in which cIAP1^Bir3^ is able to contact BTK^KD^ through a series of direct lipophilic interactions. When compared to prior BTK/cIAP1 ternary complexes^40^, this represents a fifth crystallographic snapshot, highlighting the breadth of topologies that can be achieved for even a single E3-ligase/target pair and from closely related degraders (Fig. 4D). Here, we observe a direct interaction between the cIAP1 warhead of the degrader with an outward-flipped BTK phenylalanine (F559), a residue that is mutated in an immune-impaired disease termed X-linked Agammaglobulinemia^51,52^. While this residue forms an induced contact with BCCov, it is nevertheless dispensable for degradation, consistent with flexibility in this complex and suggesting that potency might be retained even in the presence of some emerging interface mutations. Despite these intimate protein-protein contacts, the BTK~BCCov-cIAP1 ternary complex exhibits negative cooperativity. While this may involve energetic trade-offs in the conformation and entropy of the ligand, there may also be unfavorable steric clash between the C terminal Helix of cIAP1^Bir3^ and the A-loop of BTK. Modeling of this region was done cautiously, as density pertaining to both cIAP1 and BTK nearly intersect, with the A-loop of BTK appearing unstructured and potentially strained (Fig. 5B).

Finally, while BCCov and BCNC lead to potent degradation of BTK, analysis of the degradation profiles reveals an interesting observation. Of the four degrader/BTK combinations characterized (BCCov + BTK^FL^; BCCov + BTK^C481S^; BCNC + BTK^FL^; BCNC + BTKC4^C481S^), we observe a partial hook effect only for the pairing that permits covalent BTK recruitment (BCCov + wt BTK^FL^) (Fig. 1C, D, Supplementary Fig. 7B), though not in THP-1 cells with endogenous levels of BTK and cIAP1 (Supplementary Fig. 2B). This phenomenon could be intensified for covalent irreversible degraders, owing to the increase in binary occupancy afforded by the covalent bond which here may approach saturation as evidenced by our mass spectrometry titration (Supplementary Fig. 3C). While these observations are consistent with a greater hook effect for our covalent analog, the possible in vivo consequences of this effect remains unclear, due to the dynamic nature of dosing, distribution, and metabolism.

**Fig. 6:**
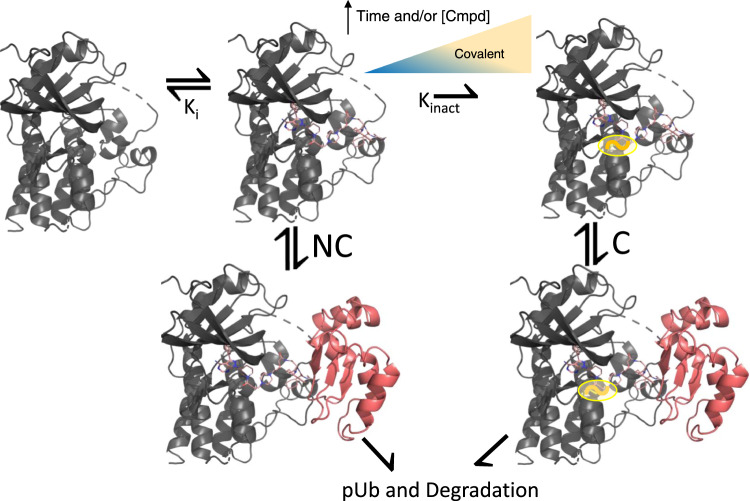
Proposed model of BTK degradation by BCCov. BTK binding to BCCov is first a reversible event K_i_. Pathway NC shows the ubiquitination and degradation of the non-covalent [BTK-BCCov] complex. Alternatively, pathway C shows the ubiquitination and degradation of a covalent [BTK~BCCov] complex that follows irreversible covalent modification (K_inact_) by BCCov. The blue/yellow coloring signifies the tendency of BTK to follow either a non-covalent or covalent degradation pathway. It is represented as a gradient as the precise contributions of each pathway may vary with exposure time and compound concentration.

Interestingly, comparing degradation profiles for both WT and C481S BTK as well as for BCCov and BCNC, reveals that, for this experimental system, covalent reactivity is not essential. However, as covalent modification increases with both time and concentration, the balance between these paths may shift for higher doses and/or longer durations of treatment. As such, we propose a model for BTK degradation by BCCov that contains pathways associated with both covalent and non-covalent recruitment of BTK, where the population of each branch is governed by experimental conditions (Fig. 6). While non-covalent binding by BCCov is likely dominant at nanomolar levels, balance may shift toward covalent recruitment as concentrations reach the micromolar range. Looking to prior studies, we see that even for potent degraders, pharmacokinetic and efficacy studies are often conducted at high multiples of cellular DC_50_^53–57^. In the case of ARV-771, PK studies show a plasma C_max_ of ~1.7 µM after a single 10 mg/kg sc dose and concentrations >2 µM in 4 of 8 rodents tested in a sub-chronic tumor growth inhibition study^53^. Similarly, micromolar exposures are also reported for the BTK degrader SJF620 at 1 mg/kg IV as well as the cIAP-directed degrader SNIPER-87 (at 10 mg/kg)^55,56^. Indeed, in vivo testing of our prior BTK degrader, PROTAC 10, gave plasma and spleen levels of ~2–3 uM following a single high-concentration subcutaneous dose^57^, suggesting that functional consequences at high concentrations are relevant factors in degrader design.

Taken together, we demonstrate that covalent target recruitment is wholly compatible with targeted protein degradation. The extent to which covalent states are populated will be governed by a variety of factors including those intrinsic to the degrader such as potency and reactivity as well as those that can be tuned through study design (ex: dose range, frequency). In the case of BCCov where covalent and non-covalent recruitment span a continuum, covalent labeling is most likely to come into play at C_max_ or in any compartments that show degrader accumulation, while non-covalent degradation may predominate at lower exposures. As such, this may represent a feature that can be exploited or avoided in future degrader designs in order to fine-tune both safety and efficacy. Extending to the family of targets that lack readily ligandable pockets, the covalent degradation pathway offers a unique opportunity to capitalize on targeted protein degradation for classically undruggable targets.

## Methods

### Plasmid design, protein expression, and purification

For cellular assays, we used a full-length BTK (aa1-659) with a C terminal Tobacco Etch Virus protease (TEV) site, a Biotinylation Acceptor Peptide (BAP) site, and a FLAG DYKDDDDK sequence. This construct was codon optimized for mammalian cell expression and cloned into a pCDNA3.1(+) (Invitrogen Cat. #V79020) using standard molecular biology techniques. Full-length cIAP1 constructs (aa1-618) had an N terminal FLAG, BAP, and TEV sequence. This sequence was optimized for insect cell (SF9) expression, though expressed well in mammalian cells, and cloned into both pCDNA3.1(+) and pCDNA 5/TO tetracycline inducible vector (Gibco Cat. #V103320). For tetracycline-inducible experiments, a 5/TO repressor plasmid, pCDNA 6/TR was co-transfected (Invitrogen Cat. #V102520) at a 1:1 ratio with the cIAP1 pcDNA 5/TO plasmid. Mutations to BTK (T474I, C481S, and F559A) were performed using a Quikchange Site-Directed Mutagenesis kit (Agilent Cat. #200518) and standard molecular biology techniques. Primer sequences are as follows: BTK^FL-F559A^ (ctccaacgaacgggggccttgctaccgacgct, agcgtcggtagcaaggcccccgttcgttggag), BTK^FL-C481S^ (ggagatagttgagtaaactgccattagccatgtactc, gagtacatggctaatggcagtttactcaactatctcc), BTK^FL-T474I^ (gccatgtactcgatgatgatgaagataggacgctgc, gcagcgtcctatcttcatcatcatcgagtacatggc).

Truncations used in both biochemistry and structural biology include cIAP1^Bir3^ (aa260-352), cIAP1^BUCR1^ (aa260-618), and BTK^KD^ (aa384-659) were expressed and purified as previously described^40^, with a brief description below. Mutation to BTK F559A was also performed using Quikchange Site-Directed Mutagenesis kit using the primers (ccagcgaacgggggccttggagccgacg, cgtcggctccaaggcccccgttcgctgg). Biotinylation was accomplished using a chloramphenicol-resistant pACYC-BirA biotin ligase in combination with 50 µM d-biotin (Invitrogen Cat. #B20656) in the terrific broth growth medium.

BTK^KD^ was purified by resuspending SF9 cells in Binding Buffer (50 mM Tris-HCl, 300 mM NaCl, 2 mM TCEP, 0.2 mM EDTA, 10% glycerol, pH = 8). Cells were lysed via microfluidizer at 18 K PSI, centrifuged at 15 Kxg for 30 min, and incubated with anti-FLAG M2 affinity resin (Millipore-Sigma, A2220). After 3 h the resin was washed with binding buffer until no protein was detected, followed by elution with 0.2 mg ml^−1^ FLAG peptide DYKDDDDK (CPC Scientific Peptide company). Elutions were pooled, cleaved overnight with TEV Protease, and resolved using Size Exclusion chromatography HiLoad 16/60 Superdex 75 column equilibrated in size exclusion buffer (20 mM HEPES, 100 mM NaCl, 2 mM TCEP and 1% glycerol, pH = 8). Samples were concentrated to 10 mg ml^−1^, frozen in liquid nitrogen, and stored at −80 °C.

cIAP1^Bir3^ and cIAP1^BUCR1^ were expressed using BL21-DE3 *Escherichia coli (*New England Biolabs) in Terrific Broth and induced with 0.4 mM IPTG. cIAP1^Bir3^ used in HSQC NMR was expressed in minimal media containing ^13^C^15^N isotopic powder (Celtone) supplemented with 5 g liter^–1^ [13 C] glucose (Cambridge Isotope Laboratories) with a final pH of 7. Cells were resuspended in binding buffer supplemented with 10 mM Imidazole, lysed and clarified as described above, and passed through a 5 mL HisTrap HP 5-ml column (GE LifeSciences). Protein was eluted using Binding Buffer supplemented with 250 mM Imidazole. When biotinylation was required, no TEV protease was added. For crystallization purposes cIAP1^Bir3^ underwent TEV cleavage overnight, and passed through a 1 mL HisTrap HP 1-mL column to remove excess TEV and uncleaved cIAP^Bir3^. Each construct was further purified using Size Exclusion chromatography as described above.

For ubiquitination reactions, UbCh5b was subcloned into a pRSET-A plasmid and purified in the same manner as cIAP1^Bir3^.

### THP-1 growth and dose-dependent degradation

Human monocytic leukemia THP-1 Cells (ATCC TIB202) were grown in RPMI 1640 complete media (Gibco Cat# 11875-093), 10% Heat Inactivated FBS (Gibco Cat# 10438026), 2 mM L-Glutamine (Gibco Cat# 25030-081), 10 mM HEPES (Lonza Cat# 17-737E). Cells were cultured to approximately 0.8 × 10^6^ cells/mL, centrifuged at 100 x *g* at 4 °C for 5 min, and resuspended in growth medium at 1.25 × 10^6^ cells/mL, and 750 µL were plated in a 24-well format. Compounds were serially diluted in DMSO and individually added to pre-warmed complete media at a 4x concentration. Subsequently, 250 µL was added to their respective well, making it a 1x concentration. Endpoint viability was ≥95%. Each experiment was performed with n = 3 independent experiments. Three datapoints are missing due to blot.

### Expi293 cellular growth and transection

Gibco Expi293 cell line Transfection Kit (Cat.# A14524) was cultured according to the manufacturers conditions. Cells were grown at 37 °C with 5% CO_2_ to 4 × 10^6^ cells/mL using an orbital shaker set to 95 RPM. Next cells were split to 2 × 10^6^ cells/mL and transfected with plasmid DNA at a 1 µg/mL final concentration in Optimem (Gibco 31985070) using the Expifectamine 293 transfection reagent according to manufacturer’s conditions. After approximately 18 h, enhancers 1 and 2 (provided with kit). Viability was typically about 85%. When cIAP1^FL^ was transfected, it was detected via α-cIAP1 antibody there was typically some autoubiquitination detected, and thus often appeared as a laddered or smeared band via western blotting. When BTK^FL^ variants were transfected, they were detected using anti-BTK antibody (D3H5).

### Expi293 dose-dependent degradation

For dose-dependent degradation assays, cells were plated in a 24-well format and placed on a separate orbital shaker set to 115 RPM. Each well was treated with prescribed compound concentrations in the same manner as with THP-1 cells, with the exception of using Expifectamine 293 growth medium in place of RPMI 1640 complete media. After approximately 18 h post-treatment cells were harvested, centrifuged at 100 x *g* at 4 °C, washed twice with ice-cold PBS, and resuspended in RIPA buffer supplemented with a Roche protease inhibitor cocktail (Cat. #11873580001), and benzonase nuclease (Millipore-Sigma Cat# 70664). Each sample was incubated on ice for 30 min with periodic vortexing and centrifuged at 15 Kxg for 20 min. Clarified lysate was harvested and quantified using a Pierce BCA Assay Kit (Cat. #23225), and 20 µg of the sample was loaded into a 4–15% Criterion TGX gel (Bio-Rad, 5678085) and transferred to a nitrocellulose membrane (Life Technologies Cat. #IB23001) using an iBlot 2 (Life Technologies Cat. #IB21001). Proteins were detected using an iBind system (Life Technologies SLF2020). For BTK^T474I^ and BTK^T474I/C481S^ we inferred ligand entry into cells via the autoubiquitination and dose-dependent degradation of cIAP1^FL,^^58^.

### Expi293 covalent modification of BTK and timepoint degradation

For covalent modification detection assays, the compound was added to Expi293 cells after enhancer treatment. At each timepoint 10 mL of cells were harvested, rapidly cooled to 4 °C in an ice bath, and centrifuged at 100 x *g* at 4 °C for 5 min. The pellet was washed twice with ice-cold PBS, snap-frozen in liquid nitrogen, and stored at −80 °C. Each pellet was resuspended in 0.1 mL ice-cold RIPA Buffer supplemented with 40 µM BCNC (to outcompete any remaining BCCov), protease inhibitor, and benzonase, and sonicated with 10 brief blasts at power 20. Lysate was centrifuged at 15 K x *g* for 20 min at 4 °C and prepared for western blotting as detailed above. When samples were evaluated for covalent adduct formation, a purification step was required. Briefly, the ~1 mL of clarified lysate was added to 9 mL of FLAG binding buffer (25 mM HEPES 7.5, 300 mM NaCl, 10% glycerol). These samples were incubated with 250 µL of anti-FLAG M2 affinity resin (Millipore-Sigma Cat. # A2220) for 3 h at 4 °C with gentle nutation. Samples were washed via gravity column filtration and eluted with 0.2 mg/ml FLAG Peptide (DYKDDDDK, CPC Scientific Peptide Company). Each sample was concentrated to 0.1 mg/mL using an Amicon Ultra-15 Centrifugal filter with a 50 kDa MWCO (Millipore-Sigma Cat. # UFC905024). Details on mass spectrometry analysis are below.

### Expi293 tetracycline-inducible cIAP1

For assays involving inducible cIAP1 expression, 18 h after compound treatment cells were treated with a final concentration of 1.0 µg/mL tetracycline (Teknova Cat. #T3325) and shaken for another 18 h. These samples were processed for western blotting as detailed above. Washout condition consisted of 4  × 10 mL PBS resuspension, centrifugation, and removal repeated for four cycles before resuspension in cellular media.

### Antibodies and chemiluminescence

BTK was detected using a 1:250 dilution of anti-BTK monoclonal antibody (Clone D3H5 Cell Signaling Technology Cat. #8547). Vinculin was detected using a 1:2,000 dilution of anti-vinculin monoclonal antibody clone EPR8185 (Abcam Cat. #129002). Anti-rabbit HRP secondary antibody was used at a dilution of 1:2,000 (Life Technologies Cat. #31460). For cIAP1 detection with either an α-cIAP1 antibody (Clone D5G9 Cell Signaling Technology Cat. #7065) or anti-FLAG conjugated to HRP (Millipore Sigma Cat. # A8592) each used at a dilution of 1:500. Chemiluminescent substrate used here was Pierce ECL Western Blotting Substrate (Cat. #32209).

### Far western blot

FLAG-purified BTK^FL^ was resolved using a 4–15% Criterion TGX Gel and transferred to a nitrocellulose membrane as described above. It was blocked for one hour using a 5% BSA (Fisher BioReagents Cat. # BP9706100) dissolved in PBS with 0.1% Tween-20 solution (PBS-T). Subsequently, a solution of 0.5 nM biotinylated cIAP1^BUCR1^ in 5% BSA/PBS-T was incubated with the membrane for 1 h with orbital rotation and washed three times for fifteen minutes with a solution of PBS-T. Lastly, the membrane was incubated with a 1:2000 dilution of Streptavidin-HRP (Thermo Scientific Cat. # ENN100) for one hour, washed, and visualized rapidly using chemiluminescence.

### Mass spectrometry

Each sample was analyzed using intact mass spectrometry under denaturing conditions using an Agilent 6530 QToF Mass Spectrometer coupled with an Agilent Infinity 1290 UPLC chromatography system. Spectra were analyzed using ChemLaunch v1.6.8 (Healex Systems), Mass Hunter V7.0 (Agilent Technologies), and BioConfirm v8.0 (Agilent Technologies). For all proteins analyzed, approximately 2 μl of 0.1–0.4 mg/ml protein sample was added to 8 μl 0.2% trifluoroacetic acid (TFA) (Sigma T6508–100 mL). Subsequently, 5 μl of each sample was injected onto the instrument. Depending on the resolution, the amount of protein loaded was altered. Theoretical versus observed molecular weight is presented in Supplementary Table 1. While we have not experimentally determined the post-translational modifications (PTMs) resulting in observed molecular weight, we believe a loss of methionine and acetylation are likely. Variations between theoretical and observed masses may also be due to the resolution of the deconvoluted spectra leading to imperfect peak identification, particularly for a protein with heterogeneous phosphorylation states. Variation of several Daltons is observed between biological replicates and individual experiments.

Lastly, the unrelated impurity that is occasionally observed is likely an artifact of the FLAG antibody immunoprecipitation experiment or a low protein concentration injection resulting in a lower signal-to-noise ratio. As it is observed with BTK^FL~C481S^ immunopurified from cells lacking BCCov treatment, we are confident it is not related to a specific covalent modification resulting from a chimeric degrader.

### Biolayer interferometry

We performed biolayer interferometry (BLI) with an OctetRED384 system (Data Acquisition Version 9.0.0.49) as described previously^40^. Briefly, biotinylated proteins (Ligands) were diluted to 12.5 μg ml–1 in Octet buffer (20 mM HEPES pH 8.0, 150 mM NaCl, 0.02% Tween-20, and 0.02% BSA). Ligands were loaded onto streptavidin biosensors (ForteBio, 18-5019) for 120 s, washed in Octet buffer for 200 s, quenched with 50 μM d-biotin (Invitrogen) for 120 s, and washed in Octet buffer for 500 s.

When cIAP1^BUCR1^ was the Ligand and BTK^KD^ was the analyte, biotinylated cIAP1 protein was dipped into 1 μM degrader for 60 s followed by a wash for 500 s in Octet buffer. When BTK^KD~BCCov^ was the ligand and cIAP1^BUCR1^ the analyte, no binary protein-degrader formation step was needed. Instead, BTK^KD^ was incubated separately with 4x molar excess BCCov until completely covalently modified (as determined by mass spectrometry). Excess compound was desalted using a Zeba Spin Desalting Column (Cat. #89882). The preformed binary complex was then dipped into varying concentrations of analyte diluted in Octet buffer for 300 s to determine k_on_, followed by a wash for 500 s to determine k_off_. Binding kinetics were calculated and plotted using BiaEvaluation Software (version 4.1.1). Cooperativity (α) was determined by calculating the ratio of binary K_d_ to ternary complex K_d_.

### Surface plasmon resonance

Experiments were performed on a Biacore^TM^ 8k instrument (GE Healthcare). Biotinylated cIAP1^Bir3^ was captured onto a streptavidin sensor chip to levels ranging from 3000–4000 RU. Compound binding experiments were performed in 10 mM HEPES, pH 7.5, 150 mM NaCl, 0.02% BSA, 0.01% P20, and 2% DMSO at 25 °C. Experiments were carried out with *n* = 4 at 6 concentrations, with the highest concentration of 37 nM and subsequent 3-fold dilutions. BCCov was injected at a flow rate of 30 µL/min, total contact time of 120 s, and dissociation times of 5000 s, using the Single Cycle Kinetics experiment. Binding responses were processed using the Biacore 8k software to zero, x-align, double reference, and correct for excluded volume effects of DMSO in the data.

Note, as shown in Supplementary Fig. 6C, a higher concentration of injectant (12 nM) showed a slower on-rate and overall response unit signal due to incomplete removal of the initial 4 nM injectant. These data suggest even with extensive washes, the slow off-rate kinetics do not permit complete removal of compound, making subsequent analysis difficult.

### Size exclusion chromatography coupled to multi-angle light scattering

SEC MALS was performed using Wyatt Technology’s Dawn Heleos-II and Optilab T-rEX. Briefly, a Shodex KW400G-4A column was pre-equilibrated in 20 mM HEPES pH 8, 100 mM NaCl, and 2 mM TCEP. Either cIAP1^BUCR1^ with BCCov, or a preformed BTK^KD~BCCov^ or BTK^KD-F559A~BCCov^ with cIAP1^BUCR1^, were incubated for 30 min at room temperature. 30 µL of each sample was injected and run at a 0.375 mL/min flow rate. ASTRA 7.1.2 software was used to analyze these data and plotted using Graphpad Prism.

### Crystallization

BTK^KD^ and cIAP1^Bir3^ were added together with BCCov in an equimolar ratio at 150 μM each. No step was taken to pre-form a covalent binary BTK^KD^~BCCov complex. The complex was centrifuged at 15,000 x *g* at 4 °C for twenty minutes, and 200 nL of this solution was added to 200 nL of well solution (of 0.1 M BTP pH 9, 4 M potassium formate, and 2% PEG MME 2 K) and incubated at room temperature in a sitting drop format. Within 3–5 days single crystals formed. Crystals were extremely difficult to reproduce, likely due to dynamic ternary complex formation kinetics combined with covalent bond formation rate.

The binary crystal structure cIAP1^Bir3^ and BCCov was obtained by adding 8 mg/mL cIAP1^Bir3^ and 1 mM BCCov, incubating for 30 min at room temperature, centrifuged at 15Kxg for 20 min at 4 °C. 200 nL of the binary complex solution was added to 200 nL of well solution (0.17 M Sodium Acetate, 0.085 M TrisBase pH 8.5, 25.5% w/v PEG 4000, 15% v/v glycerol). After approximately 3 days single crystals grew. These crystals were easily reproduced.

### X-ray structural determination and model building

Data were collected at the SLS and at the IMCA-CAT at the Advanced Photon Source and were solved by molecular replacement using Phaser^59^ with 4KMN^60^ and 5P9J^49^ used as search models for cIAP1^Bir3^ and BTK^KD^, respectively. Refinement was primarily conducted using autoBUSTER^61^ and Coot^62^, and figures were generated in PyMOL (version 1.243pre; Schrödinger). The proteins will be deposited to the protein data bank with accession codes cIAP1^Bir3^-BCCov (8DSF) and cIAP1^Bir3^-BCCov-BTK^KD^ (8DSO) with 0% Ramachandran outliers respectively. Anisotropic data was utilized to obtain the most information possible and generate accurate models.

### NMR spectroscopy

Preparation of [^15^N,^13^C]- labelled cIAP1^Bir3^ was described previously^40^. Briefly, final sample conditions were 50 μM IAP1^Bir3^, 50 μM BCCov in 50 mM Tris, 150 mM NaCl, 2 mM TCEP, pH 7.5, 10% (v/v) D_2_O (Cambridge Isotopes Laboratories), in the presence and absence of 50 μM BTK^KD^. All NMR data were collected at 25 °C on an AVANCE 600 spectrometer (Bruker, Billerica MA) equipped with a 1.7-mm TCI Micro-Cryoprobe with a shielded z-gradient coil. The 2D [^15^N,^1^H]-HSQCs were recorded on a Nyquist grid of 64(t_1_) × 1,024(t_2_) complex points, t_1max_  =  23 ms, t_2max_  =  122 ms and 64 scans per increment. All NMR datasets were processed using TopSpin 3.5 (Bruker) and analyzed in CARA (www.cara.nmr.ch) Before processing, the datasets were zero-filled and multiplied with a cosine function in both dimensions. The backbone assignments for cIAP1^Bir3^ were described previously^40^.

### Chemical cross-linking

Cross-linking was performed as previously described^40^. Briefly, 20 µM cIAP1^BUCR1^, 20 µM BTK^KD^, 20 µM BTK^KD~BCCov^, and 20 µM BC5P^40^ were incubated in respective combinations (Supplementary Fig. 5C) for 30 min. Next either DMSO or 20 µM BMOE (Thermo Scientific Cat. #22323) was added and allowed to incubate for 15 min, quenched with 1 mM DTT, and resolved using a 4–15% Criterion TGX Gel.

### In vitro ubiquitination and degradation

Recombinant BTK^FL^ was ubiquitinated using a Ubiquitylation Assay Kit according to the manufacturers' conditions (ab139467) with some modifications. Here, 300 nM of BTK^FL^ or BTK^FL^~BCCov was incubated with the ubiquitylation reaction containing 300 nM cIAP1^BUCR1^. The reaction was performed at 37 °C for 2 h. The reaction was quenched by adding 20 µM PYR41 E1 inhibitor on ice for 30 min. Ubiquitination was confirmed by western blotting using 1:1000 dilution of anti-BTK monoclonal antibody (Clone D3H5 Cell Signaling Technology Cat. #8547) and anti-rabbit HRP secondary antibody was used at a dilution of 1:2,000 (Life Technologies Cat. #31460).

Degradation reactions were performed using Expi293 clarified lysate prepared as previously described^63^ with some modifications. Briefly, Expi293 cells, pre-transfected with cIAP1^FL^ from a pCDNA3.1 plasmid, were diluted to 2 × 10^6^ cells/mL in a total of 50 mL. Cells were washed twice in ice-cold PBS and resuspended in 1 mL proteasomal lysis buffer (50 mM HEPES 7.5, 10 mM NaCl, 1.5 mM MgCl_2_, 1 mM EDTA, 1 mM EGTA, 250 mM sucrose, 5 mM DTT). The sample was sonicated twice for 10 s at power 20, followed by centrifugation at 4 °C at 17Kxg for 30 min. Clarified lysate was stored at −80 °C. Degradation assays were performed by adding 5 µl ubiquitination assay reaction mixture, 12.5 µl clarified lysate, and 25 µl proteasomal lysis buffer supplemented with 5 mM ATP. Proteasomal dependence was determined by adding 50 µM MG132 (Millipore-Sigma #474790) or equivalent amounts of DMSO. After 30 min reactions were stopped by adding SDS sample buffer and boiling at 95 °C for 10 min. Total ubiquitinated BTK was detected through western blotting using a 1:250 dilution of anti-BTK monoclonal antibody (D3H5) and 1:1000 dilution of anti-rabbit HRP. Over time high molecular weight species decrease over time, potentially due to dUB activity, uninhibited proteasomal activity, or BTK^FL^ instability over time. However, MG132-dependent degradation was observed and easily reproducible.

Kinase domain ubiquitination reactions were performed as described previously^40^. Briefly, 5 µM of preformed binary complex, biotinylated BTK^KD~BCCov^, was mixed with 0.3 µM E1 (Boston Biochem Cat. #E300), 0.85 µM UbcH5b, and 50 µM ubiquitin (Millipore-Sigma Cat# U5382), and a buffer comprised of 20 mM Tris-HCl pH 7.5, 50 mM NaCl, 5 mM ATP, 2 mM MgCl_2_ and 2 mM DTT. Subsequently, 5 µM, 1.67 µM, 0.56 µM, 0.19 µM, or 0.062 µM cIAP1^BUCR1^ was added to each reaction mixture, incubated for 15 min at room temperature, and quenched with 5 mM EDTA pH 8. Ubiquitination of BTK^KD^ was resolved using a streptavidin-HRP and Chemiluminescent substrate with the iBind and iBlot systems as described above.

### Statistics and reproducibility

Dose-dependent Degradation was analyzed using ImageJ (NIH, version 1.50i). Densiometric analysis was performed, normalized to DMSO signal, and analyzed using GraphPad Prism (Version 9.0.0 (121)). Non-linear fit one-phase decay analysis was used to obtain DC_50_ values. When we observed a hook effect, some points were excluded as part of the fit. In Supplementary Fig. 7B the complete curve was fit using GraphPad Prism non-linear regression bell-shaped curve where X is the concentration. However, using this method we were unable to obtain stable values for our DC_50._ SEM error was plotted for each point. When determining statistically significant differences, we used a two-sided student’s unpaired T-Test.

The number of times each experiment was performed is listed in the corresponding figure legend.

### Synthesis and characterization of substrates

See Supplementary Information.

### Reporting summary

Further information on research design is available in the Nature Portfolio Reporting Summary linked to this article.

## Supplementary information

Supplementary Information

Reporting Summary

## Data Availability

The data that support the findings of this study are available within the main text and its Supplementary Information file. The structural coordinates from X-ray crystallography experiments have been deposited in the RCSB PDB database with the following accession codes: cIAP1^Bir3^-BCCov (8DSF) and cIAP1^Bir3^-BCCov-BTK^KD^ (8DSO). Source Data provided as Source Data file. Data is also available from the corresponding author upon request. Source data are provided with this paper.

## Source data

Source Data
