# Supplementary material for: A covalent BTK ternary complex compatible with targeted protein degradation
Source: Nat Commun. 2023 Mar 2;14:1189. doi: 10.1038/s41467-023-36738-z (PMC9981747; doi:10.1038/s41467-023-36738-z)
Supplement: Supplementary file 1 — Supplementary Information [file 41467_2023_36738_MOESM1_ESM.pdf]

Supplementary Information:

A Covalent BTK Ternary Complex Compatible with Targeted Protein Degradation

James Schiemer[1], Andrew Maxwell[1], Reto Horst[1], Shenping Liu[1], Daniel P. Uccello[1], Kris Borzilleri[1], Nisha Rajamohan[1], Matthew F. Brown[1], Matthew F. Calabrese[1]*

Affiliations

[1] Discovery Sciences, Pfizer Worldwide Research and Development, Groton, CT, USA.

Corresponding Author:

Correspondence to Matthew F Calabrese

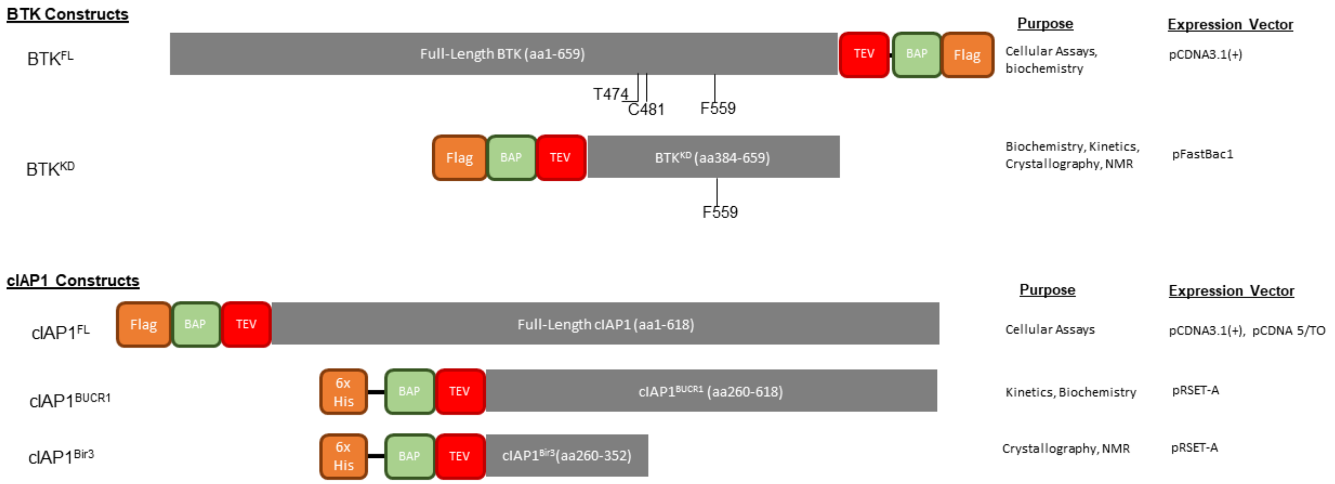

Supplementary Figure 1: Plasmid Construct Information
Information pertaining to cIAP1 and BTK constructs used throughout the manuscript, tags, points for mutagenesis, truncations, and expression vectors. Not drawn to scale.

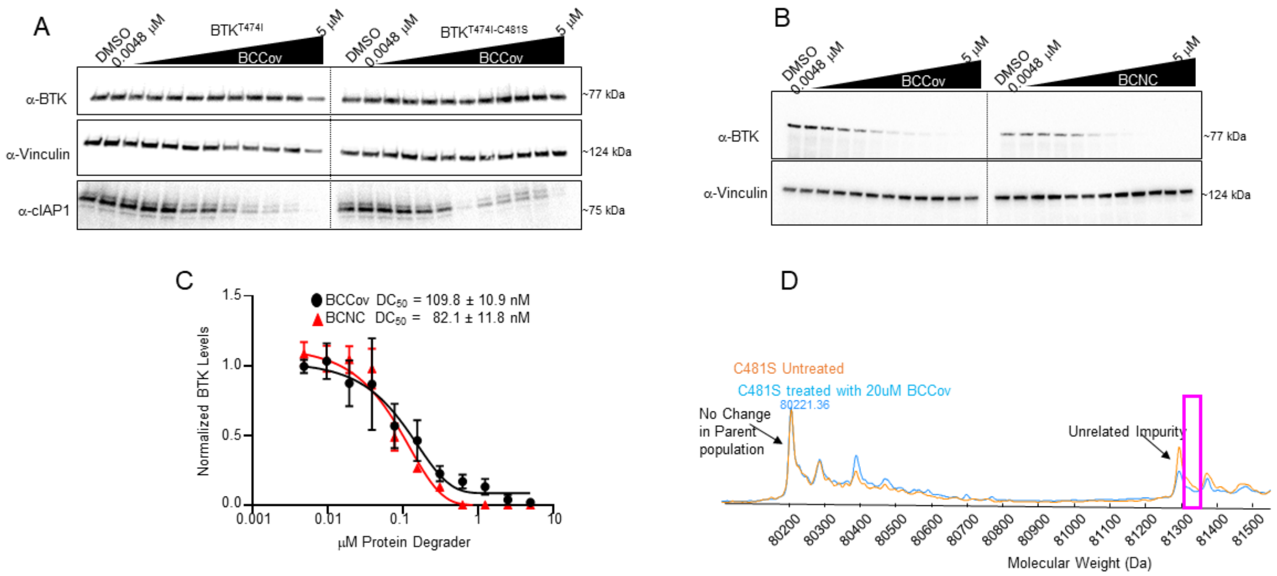

Supplementary Figure 2: BTK Gatekeeper Mutagenesis and THP-1 Degradation

a, Dose dependent degradation of BTK^{FL} mutants (T474I, T474I/C481S) via BCCov, transiently expressed in Expi293 cells with cIAP1^{FL}. Below are corresponding vinculin loading control blots, and cIAP1^{FL} degradation. The western blot is a representative blot from 3 independent experiments. **b,** Representative western blots of endogenous BTK degradation in THP-1 cells by BCNC and BCCov with corresponding vinculin loading controls. The western blot is a representative blot from 3 independent experiments. **c,** Analysis of degradation studies in THP-1 cells with BCCov (black circles) and BCNC (red triangles). Each compound was tested in 3 separate experiments with the mean value plotted, and error bars reflecting the standard error of the mean (SEM). **d,** Representative intact mass spectrometry chromatograph of Immunopurified BTK$^{FL-C481S}$ from Expi293 cells pre-treated with 20 µM BCCov (blue) or no treatment (orange). The magenta box indicates location of peaks corresponding to the expected, but not observed, molecular weight C481S-BCCov covalent complex. These data are representative of 3 individual experiments. Samples processed from different blots were from the same experiment, and processed in parallel using DMSO treatment as a normalization control.

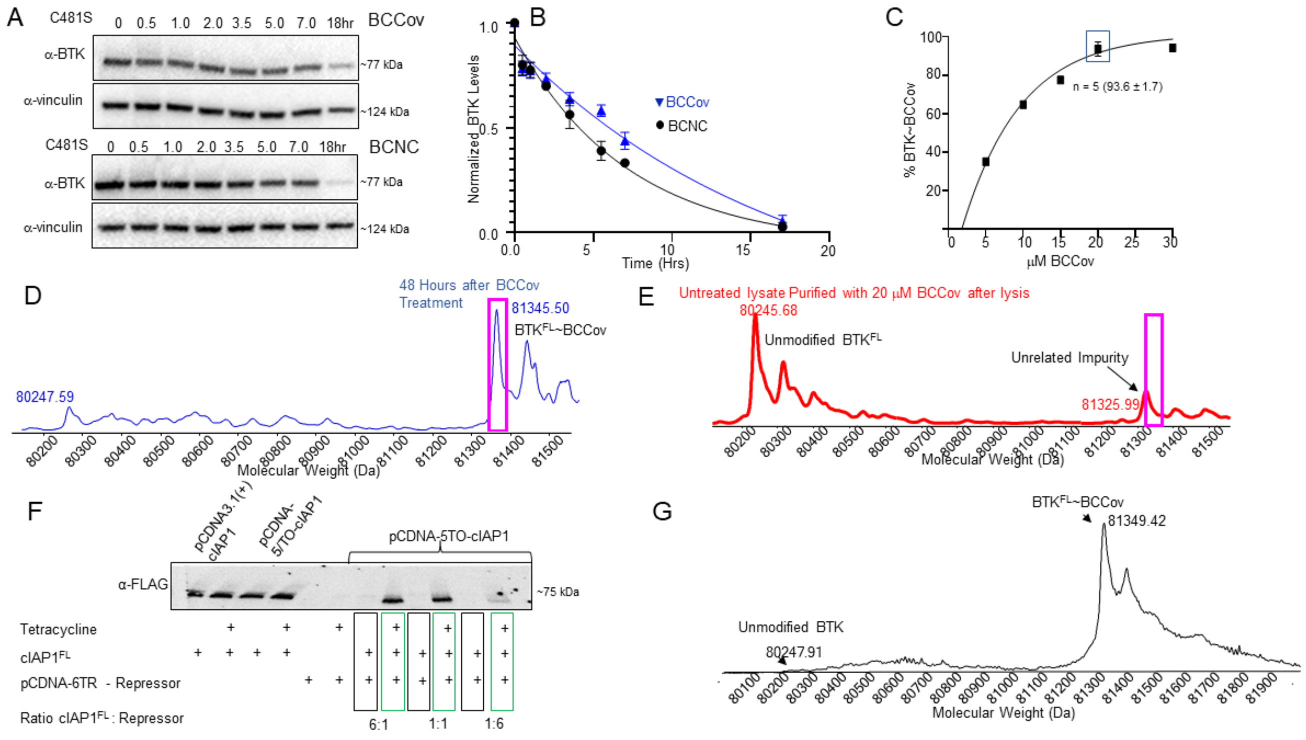

Supplementary Figure 3: Establishing an Inducible cIAP1-BTK Degradation System

a, Time dependent degradation of $BTK^{FL\text{-}C481S}$ with BCCov or BCNC **b,** BCCov quantification from 3 independent experiments is shown as blue triangles, while BCNC is plotted with black circles; each with SEM error bars. **c,** Single data points (n = 1 for 5, 10, 15, and 30 µM) showing the percent BTK^{FL} covalently modified by BCCov after 18 hour BCCov treatment. Quantitation was derived from area under the curve for the spectral traces of each species (modified and unmodified BTK). The 20 µM point has an experimental n = 5 plotted with error bars reflecting SEM. **d,** Intact mass spectrometry chromatograph showing covalent modification of BTK^{FL} persists 48 hours after a single dose of 20 µM BCCov. The magenta box indicates the population of BTK^{FL}~BCCov. These are representative data from 3 independent experiments. **e,** Representative intact mass spectrometry chromatograph of naïve BTK^{FL} (cells without BCCov treatment) and subsequently supplemented with 20 µM BCCov during the immunopurification step to account for potential non-specific labelling of BTK^{FL}. These data are representative of 3 independent experiments.

f, Detection of transiently transfected $cIAP1^{FL}$ in pCDNA3.1(+) and 5/TO with or without a repressor plasmid (6TR) co-transfected at varying molar ratios, with or without tetracycline. This western blot is representative of 3 independent experiments. **g,** Intact mass spectrometry chromatograph of purified BTK^{FL} subsequently treated with BCCov until approximately 100% labelled and desalted to remove excess compound. These are representative data from 3 independent experiments.

Samples processed from different blots were from the same experiment and processed in parallel using DMSO treatment as a normalization control.

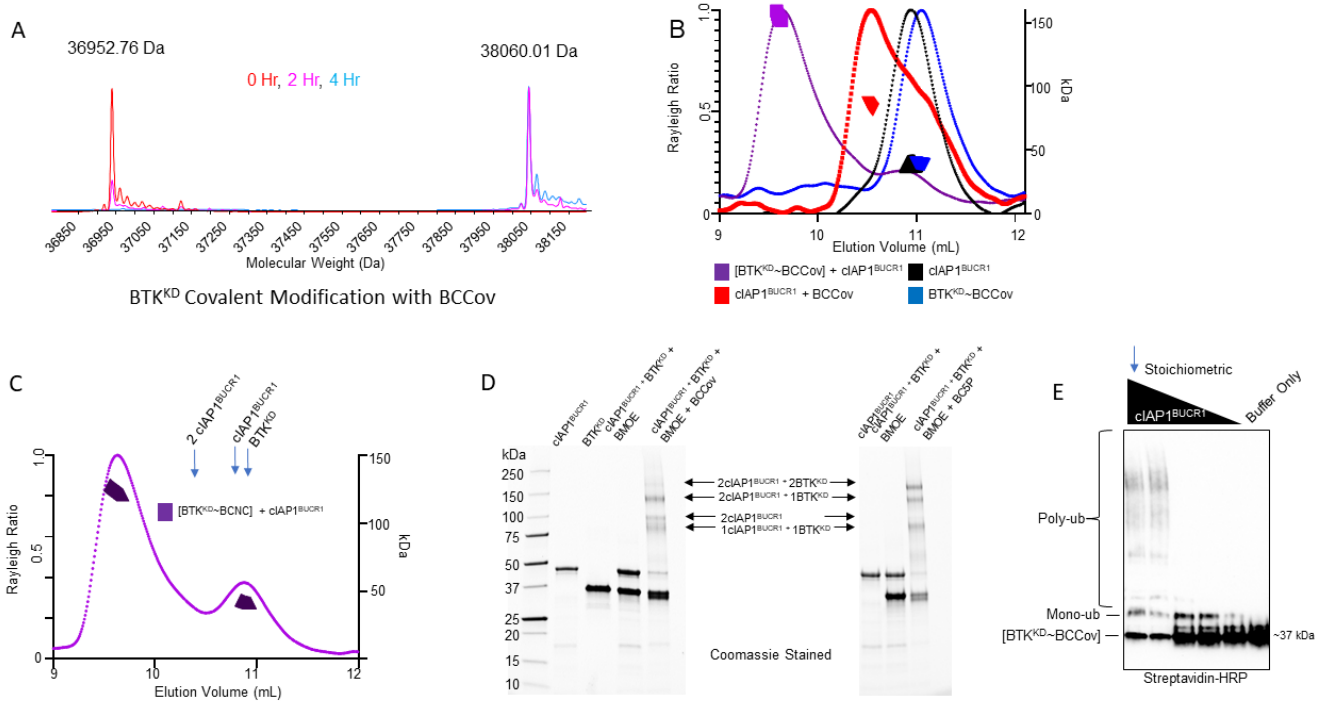

Supplementary Figure 4: BCCov Promotes a multi-component BTK-cIAP1 complex

a, Intact mass spectrometry chromatograph showing BTK^{KD} modification via in vitro BCCov treatment at a 0-, 2-, or 4-hour time points colored as red, magenta, and blue respectively. These data are representative of 3 independent experiments.

b, SEC MALS chromatograph of BTK^{KD}~BCCov (blue), $cIAP1^{BUCR1}$ (black), $cIAP1^{BUCR1}$ dimer after incubation with BCCov (red), and an oligomeric BTK^{KD}, $cIAP1^{BUCR1}$, BCCov complex (purple). Rayleigh ratio shown as dotted lines (left axis) and calculated molecular weights are shown in block-fill (right axis) **c,** SEC MALS profile demonstrating high molecular weight $cIAP1^{BUCR1}$, BTK^{KD}, BCNC complex. Rayleigh ratio shown as dotted lines (left axis) and calculated molecular weights are shown in block-fill (right axis). Known species elution volumes are noted with blue arrows. Data for SEC MALS figures b and c are representative of 3 independent experiments. **d,** Chemical cross-linking of BTK^{KD} and $cIAP1^{BUCR1}$ with a homo-bifunctional maleimide cross-linker (BMOE) in the presence of BCCov or BC5P[40]. **e,** *In vitro* ubiquitination of pre-formed BTK^{KD}~BCCov with varying concentrations of $cIAP1^{BUCR1}$. Polyubiquitinated species detected by biotinylated BTK^{KD} via streptavidin-HRP. Data from figures **d** and **e** are representative of 3 independent experiments.

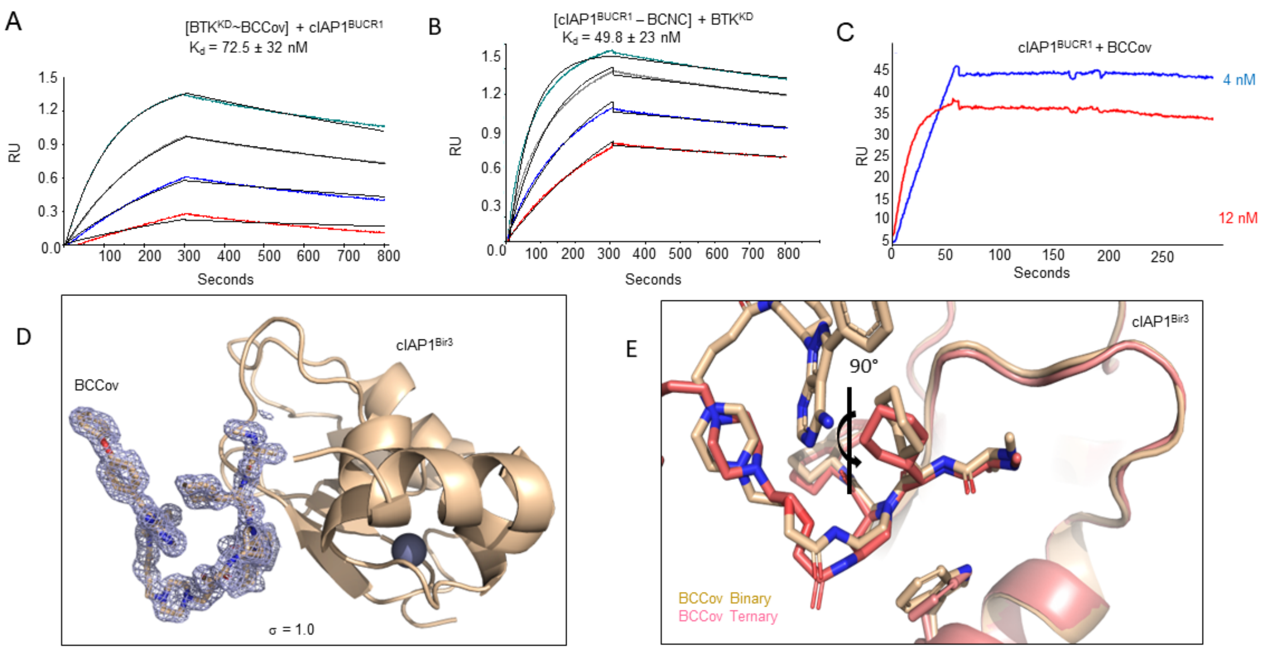

Supplementary Figure 5: Biophysical and Structural Characterization of Binary and Ternary Complexes **a,** Representative BLI chromatograph of preformed biotinylated BTK^{KD}~BCCov with varying concentrations of $cIAP1^{BUCR1}$ (3 μM green, 1.5 μM gray, 0.75 μM blue, 0.375 μM red). **b,** BLI Chromatograph of $cIAP1^{BUCR1}$-BCNC forming a ternary complex with BTK^{KD} (3 μM green, 1.5 μM gray, 0.75 μM blue, 0.375 μM red). For BLI experiments, K_d values were determined from three independent experiments with at least 4 points contributing to a single mean value. **c,** Representative SPR chromatograph demonstrating BCCov (blue) binding to immobilized biotinylated-$cIAP1^{BUCR1}$. The second trace (red) is a sequential higher dose injection of BCCov. These are representative traces from 3 independent experiments. **d,** Binary crystal structure of $cIAP1^{Bir3}$ and BCCov, with ligand 2Fo-Fc electron density contoured to an RMSD σ = 1.0. **e,** Overlay of a $cIAP1^{Bir3}$ in a binary complex (wheat) or ternary complex (salmon) with BCCov. The IAP ligand warhead cyclohexyl group rotates approximately 90° between each pose.

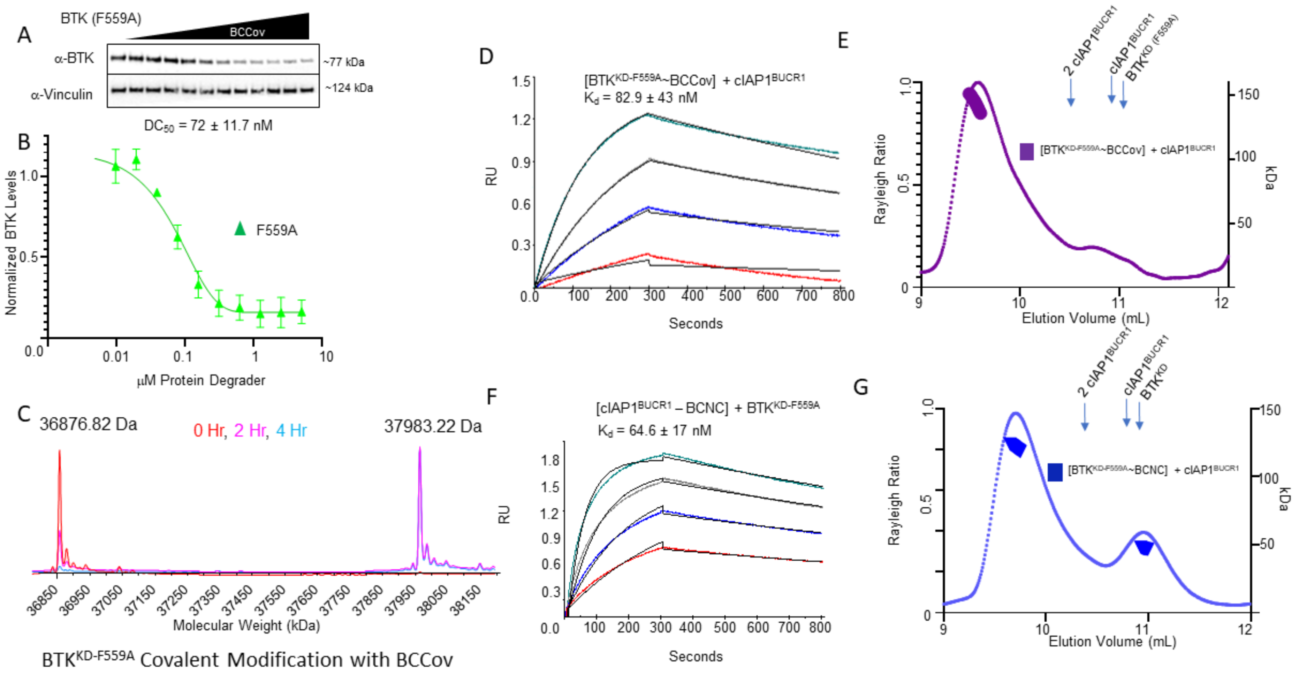

Supplementary Figure 6: BTK^{F559} is Dispensable for Productive Ternary Complex Formation and Degradation

a, b Representative western blot of Expi293 cell dose dependent degradation of BTK$^{FL\text{-}F559A}$ and densiometric analysis of 3 separate experiments below with error plotted as SEM. **c,** Intact mass spectrum chromatograph showing BTK$^{KD\text{-}F559A}$ modification via BCCov at a 0-, 2-, or 4-hour time points colored as red, magenta, and blue respectively. **d,** BLI chromatograph of a BTK$^{KD\text{-}F559A}$~BCCov preformed binary complex forming a ternary complex with cIAP1^{BUCR1} (3 µM green, 1.5 µM gray, 0.75 µM blue, 0.375 µM red). K_d value was determined from three independent experiments with at least 4 points contributing to a single mean value. **e,** SEC MALS profile demonstrating high molecular weight cIAP1^{BUCR1} and BTK$^{KD\text{-}F559A}$~BCCov complex. Rayleigh ratio shown as dotted lines (left axis) and calculated molecular weights are shown in block-fill (right axis) **f,** BLI Chromatograph of cIAP1^{BUCR1}-BCNC forming a ternary complex with BTK$^{KD\text{-}F559A}$. **g,** SEC MALS profile demonstrating high molecular weight cIAP1^{BUCR1}, BTK$^{KD\text{-}F559A}$, BCNC complex. Rayleigh ratio shown as dotted lines (left axis) and calculated molecular weights are shown in block-fill (right axis) Known species elution volumes are noted with blue arrows.

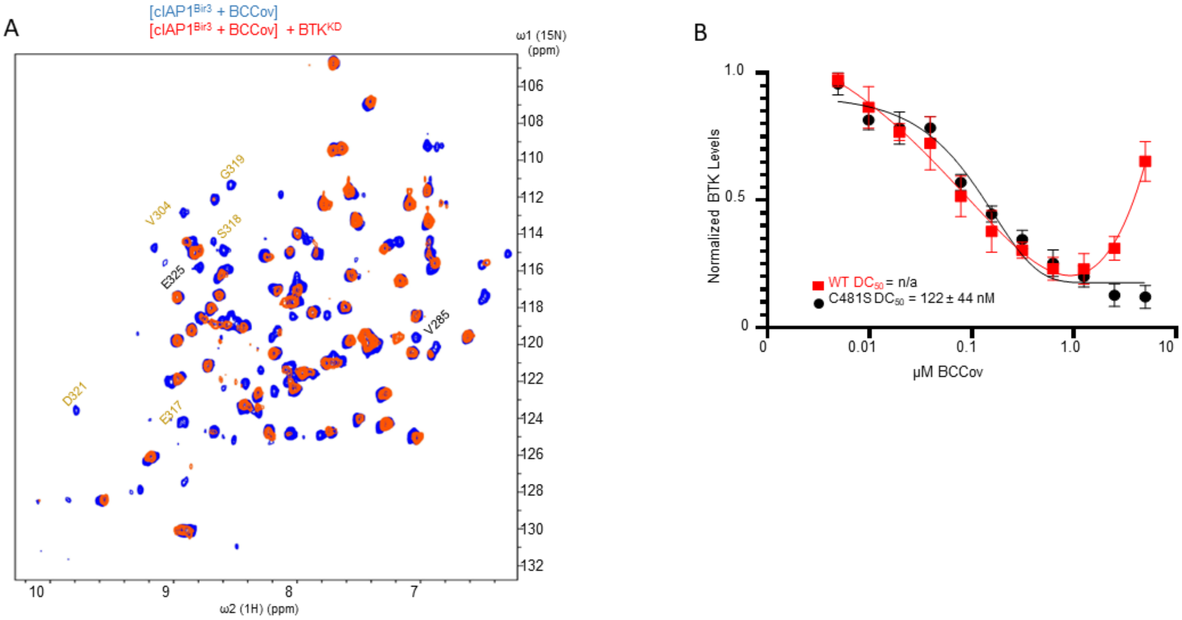

Supplementary Figure 7: 2D NMR Ternary Complex Analysis

a, Overlay of 2D [^{15}N,^{1}H]-HSQC NMR spectra of uniformly [^{15}N,^{13}C]- labelled cIAP1^{Bir3}– BCCov in absence and presence of BTK^{KD} are shown in blue and red, respectively. cIAP1^{Bir3} signals that are broadened beyond detection in presence of BTK^{KD} are highlighted in orange, and those that experience large chemical shift perturbations are highlighted in black. **b,** Dose dependent BTK Degradation profile from Figure 1c in which the entire curve was fit. Quantification and DC_{50} plot for degradation of BTK^{FL} or BTK$^{FL-C481S}$ across 3 separate experiments shown in panel. Mean values were plotted with SEM Error bars.

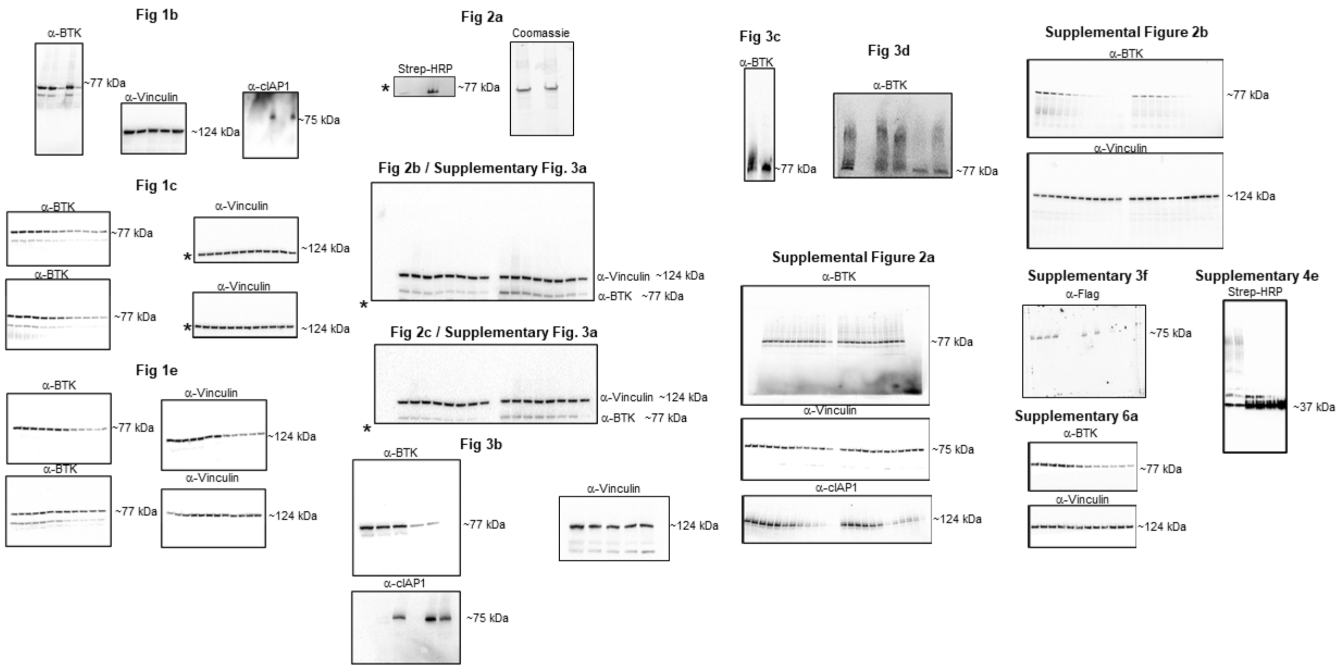

Supplementary Figure 8: Uncropped Western Blots
Western blot data for all figures without cropping. An asterisk (*) is placed in the instances where the membrane was cut to divide a blot by molecular weight or manually remove overblown saturated pixels unrelated to the protein of interest.

Supplementary Table 1: Molecular Weights and Modifications

These data describe the theoretical and experimentally observed molecular weights with a change in mass (Δ) listed. The PTM column describes predicted modifications that explain the Δ mass shift. The covalent modification column refers to the theoretical and observed molecular weight increase after BTK forms a covalent bond with BCCov.

Construct / Compound	Theoretical MW (Da)	Observed MW (Da)	Predicted PTM(s) (Da)	Predicted MW after Covalent Modification (Da)	Observed MW after Covalent Modification (Da)
BCCov	1106.36				
BAP-FLAG-6xHis-TEV-BTK^{KD}	36815.84	36952.76 (Δ136.92)	-DesMet (-131.19 Da), +Acetylation (+42.0373), +Biotinylation (226.03) = -136.88 (Δ0.04)	38059.59	38060.98 (Δ1.39)
BAP-FLAG-6xHis-TEV-$BTK^{KD\text{-}F559A}$	36739.74	36876.82 (Δ137.08)	-DesMet (-131.19 Da), +Acetylation (+42.0373), +Biotinylation (226.03) = -136.88 (Δ0.2)	37983.18	37983.22 (Δ0.04)
FLBTK-TEV-BAP-FLAG	80324.49	80245.68 (Δ78.81)	-DesMet (-131.19 Da), +Acetylation (+42.0373 Da) = -89.15 (Δ10.34)	81352.04	81345.50 (Δ6.54)
$FLBTK^{C481S}$ -TEV-BAP-FLAG	80308.43	80221.36 (Δ87.07)	-DesMet (-131.19 Da), +Acetylation (+42.0373 Da) = -89.15 (Δ2.08)	81327.72	None.

(Δ = Theoretical vs observed difference)

Supplementary Table 2: Crystallographic Summary

Statistical summary of crystal structures. Anisotropic data was used for each crystal structure determined and is reflected in the reported statistics.

	cIAP1^{Bir3}-BCCov	BTK^{KD}-BCCov-cIAP1^{Bir3}
**Data collection**		
Space group	P1	P321
Cell dimensions		
a, *b*, *c* (Å)	28.99 30.77 118.63	104.36 104.36 110.29
α, β, γ (°)	96.70 90.22 111.08	90.0 90.0 120.0
Resolution (Å)	1.391(1.391-1.495)*	2.337(2.337-2.734)*
R_{pim}	0.037(0.452)	0.113(0.551)
I / σI	10.6(1.9)	4.7(1.6)
Completeness (%)	72.4(27.3)	88.9(41.2)
Redundancy	1.8(1.6)	9.9(9.1)
**Refinement**		
Resolution (Å)	1.5	2.334
No. reflections	47320	17804
R_{work} / R_{free}	0.1817/0.2084	0.2423/0.2918
No. atoms		
Protein	2885	2683
Ligand/ion	242	82
Water	377	72
B-factors		
Protein	20.15	72.13
Ligand/ion	15.52	53.93
Water	35.82	58.77
R.m.s. deviations		
Bond lengths (Å)	0.008	0.008
Bond angles (°)	0.81	0.93

Each structure was solved from one crystal using Anisotropic Data. *Values in parentheses are for highest-resolution shell.

Supplementary Synthetic Methods:

Primary vendors for chemicals used in synthetic strategies include Sigma-Aldrich, Combi-Blocks, Fisher Scientific and Oakwood Chemical. Data were gathered on a Waters Acquity LC/MS with single quadrupole mass detector (Waters Corporation, Milford MA) and ZSpray dual ESI/APCI ionization source. The LC instrument includes a CTC Pal autosampler (Trajan Scientific and Medical, Melbourne, Australia), and Waters Acquity binary pump, column manager, photodiode array detector, and evaporative light scattering detector. The instrument acquisition and data handling were done with Waters MassLynx and OpenLynx software version 4.1. Stepwise synthetic methods are described in detail following the schemes below.

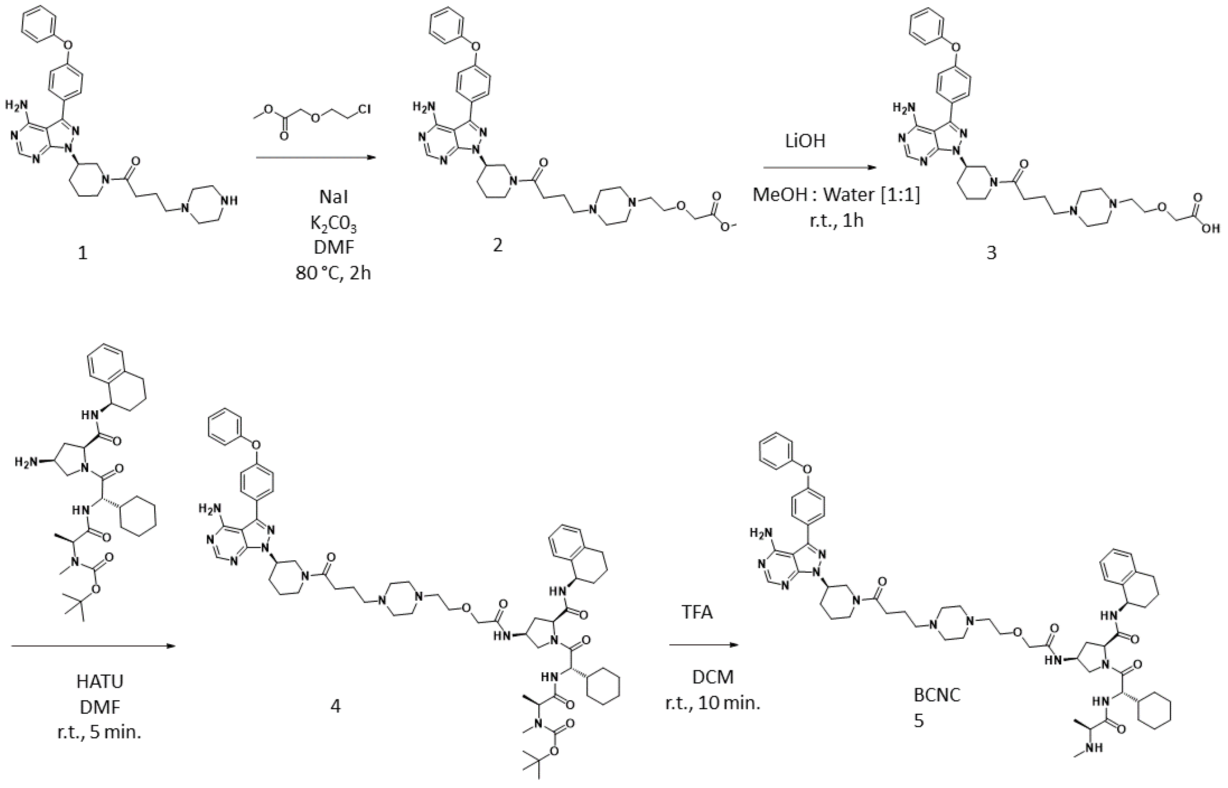

(*R*)-1-(3-(4-amino-3-(4-phenoxyphenyl)-1*H*-pyrazolo[3,4-d]pyrimidin-1-yl)piperidin-1-yl)-4-(piperazin-1-yl)butan-1-one (1) was prepared according to a literature procedure from Tinworth et al[1].

methyl (*R*)-2-(2-(4-(4-(3-(4-amino-3-(4-phenoxyphenyl)-1*H*-pyrazolo[3,4-d]pyrimidin-1-yl)piperidin-1-yl)-4-oxobutyl)piperazin-1-yl)ethoxy)acetate (2): Compound **1** (140 mg, 0.26 mmol) and methyl 2-(2-chloroethoxy)acetate (79 mg, 0.52 mmol) were dissolved in DMF (4 mL). The potassium carbonate (107 mg, 0.78 mmol) and sodium iodide (78 mg, 0.52 mmol) were added and the reaction was brought to 80 °C overnight. The reaction was cooled to room temperature and extracted between EtOAc and a brine-water [1:1] solution. The organic layer was dried over sodium sulfate and evaporated to yield Compound **2** as a crude oil. *m/z* calcd for $[M+H^+]$ 657.3, found 657.7.

(*R*)-2-(2-(4-(4-(3-(4-amino-3-(4-phenoxyphenyl)-1*H*-pyrazolo[3,4-d]pyrimidin-1-yl)piperidin-1-yl)-4-oxobutyl)piperazin-1-yl)ethoxy)acetic acid (3): Compound **2** (170mg, 0.26 mmol) was dissolved in methanol (10 ml). The lithium hydroxide (62 mg, 2.59 mmol) and water (10 mL) were added and the reaction was allowed to stir at room temperature for 1 hour. The reaction was evaporated to 2/3 the original volume to remove bulk methanol, and the solution was adjusted to pH ~ 7 with saturated aqueous sodium hydrogen sulfate solution. The resulting solution was extracted with n-butanol (30 mL). The

organic layer was dried over sodium sulfate and evaporated to yield Compound **3** as an off-white solid. *m/z* calcd for [M+H+] 643.3, found 643.6.

***tert*-butyl ((*S*)-1-(((*S*)-2-((2*S*,4*S*)-4-(2-(2-(4-(4-((*R*)-3-(4-amino-3-(4-phenoxyphenyl)-1*H*-pyrazolo[3,4-d]pyrimidin-1-yl)piperidin-1-yl)-4-oxobutyl)piperazin-1-yl)ethoxy)acetamido)-2-(((*R*)-1,2,3,4-tetrahydronaphthalen-1-yl)carbamoyl)pyrrolidin-1-yl)-1-cyclohexyl-2-oxoethyl)amino)-1-oxopropan-2-yl)(methyl)carbamate (4):** Compound **3** (75 mg, 0.12 mmol) was dissolved in DMF (1 mL). The *tert*-butyl ((S)-1-(((S)-2-((2S,4S)-4-amino-2-(((R)-1,2,3,4-tetrahydronaphthalen-1-yl)carbamoyl)pyrrolidin-1-yl)-1-cyclohexyl-2-oxoethyl)amino)-1-oxopropan-2-yl)(methyl)carbamate (68 mg, 0.12 mmol) was added, followed by HATU (49 mg, 0.13 mmol), and the reaction was stirred at room temperature for 5 minutes. The reaction was extracted between ethyl acetate (20 mL) and water (100 mL). The organic layer was washed with brine, dried over sodium sulfate, and evaporated to yield Compound **4** as a white solid. *m/z* calcd for [M+H+] 1208.7, found 1209.4.

(2*S*,4*S*)-4-(2-(2-(4-(4-((*R*)-3-(4-amino-3-(4-phenoxyphenyl)-1*H*-pyrazolo[3,4-d]pyrimidin-1-yl)piperidin-1-yl)-4-oxobutyl)piperazin-1-yl)ethoxy)acetamido)-1-((*S*)-2-cyclohexyl-2-((*S*)-2-(methylamino)propanamido)acetyl)-*N*-((*R*)-1,2,3,4-tetrahydronaphthalen-1-yl)pyrrolidine-2-carboxamide (BCNC - 5): Compound **4** (90 mg, 0.074 mmol) was dissolved in DCM (2 mL). The TFA (1 mL) was added and the reaction was stirred at room temperature for 10 minutes. The reaction was evaporated to a crude and purified by LC-prep to afford the final product (BCNC). *m/z* calcd for [M+H+] 1108.6, found 1109.3.

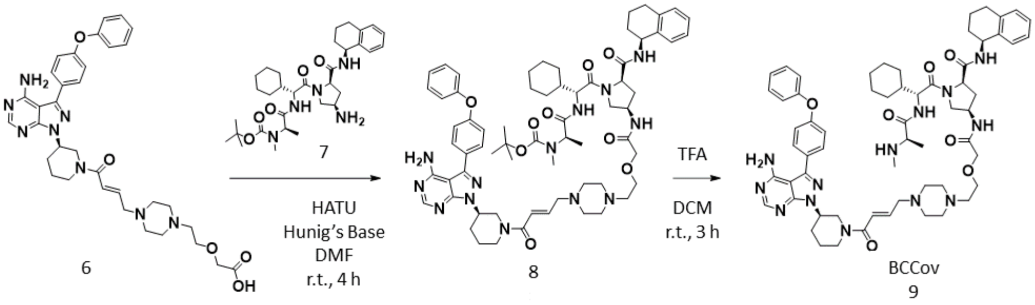

(*R,E*)-2-(2-(4-(4-(3-(4-amino-3-(4-phenoxyphenyl)-1H-pyrazolo[3,4-d]pyrimidin-1-yl)piperidin-1-yl)-4-oxobut-2-en-1-yl)piperazin-1-yl)ethoxy)acetic acid (6) was prepared according to a literature procedure from Tinworth et al[1].

***tert*-butyl ((*R*)-1-(((*R*)-2-((2*R*,4*R*)-4-(2-(2-(4-((*E*)-4-((*R*)-3-(4-amino-3-(4-phenoxyphenyl)-1*H*-pyrazolo[3,4-d]pyrimidin-1-yl)piperidin-1-yl)-4-oxobut-2-en-1-yl)piperazin-1-yl)ethoxy)acetamido)-2-(((*S*)-1,2,3,4-tetrahydronaphthalen-1-yl)carbamoyl)pyrrolidin-1-yl)-1-cyclohexyl-2-oxoethyl)amino)-1-oxopropan-2-yl)(methyl)carbamate (8):** Compound **6** (200 mg, 0.31 mmol) was dissolved in DMF (4 mL). HATU (178 mg, 0.47 mmol) was added and the solution was stirred at room temperature for 30 minutes. After 30 minutes, a solution of Comppound **7** (200 mg, 0.34 mmol) and Hunig's base (161 mg, 1.25 mmol) in DMF (1 mL) was added to the reaction mixture. The reaction was stirred at room temperature for an additional 4 hours. The reaction was poured into water (60 mL) and was extracted with ethyl acetate (80 mL x 2). The combined organic layers were washed with brine, dried over sodium sulfate, evaporated, and purified via silica chromatography to yield Compound **8** as a pale yellow gum. *m/z* calcd for [M+Na+] 1228.7, found 1229.3.

(2*R*,4*R*)-4-(2-(2-(4-((*E*)-4-((*R*)-3-(4-amino-3-(4-phenoxyphenyl)-1H-pyrazolo[3,4-d]pyrimidin-1-yl)piperidin-1-yl)-4-oxobut-2-en-1-yl)piperazin-1-yl)ethoxy)acetamido)-1-((*R*)-2-cyclohexyl-2-((*R*)-2-

(methylamino)propanamido)acetyl)-*N*-((*S*)-1,2,3,4-tetrahydronaphthalen-1-yl)pyrrolidine-2-carboxamide (BCCov - 9): Compound **8** (100 mg, 0.083 mmol) was dissolved in DCM (4 mL). TFA (0.5 mL) was dropwise added and the mixture was stirred at room temperature for 3 hours. The reaction mixture was concentrated and the residue was dissolved in acetonitrile : water (3 mL : 1 mL). The solution was basified with aqueous sodium bicarbonate solution to pH ~ 9. The suspension was concentrated and was purified via LC-prep to afford the final product (BCCov). *m/z* calcd for $[M+H^+]$ 1106.6, found 1107.0.

Supplementary References:

1. Tinworth, C.P. et al. PROTAC-Mediated Degradation of Bruton's Tyrosine Kinase Is Inhibited by Covalent Binding. *ACS Chemical Biology* **14**, 342-347 (2019).
