# Supplementary material for: A covalent BTK ternary complex compatible with targeted protein degradation
Source: Nat Commun. 2023 Mar 2;14:1189. doi: 10.1038/s41467-023-36738-z (PMC9981747; doi:10.1038/s41467-023-36738-z)
Supplement: Supplementary file 2 — Reporting Summary [file 41467_2023_36738_MOESM2_ESM.pdf]

nature portfolio

Corresponding author(s): Matthew Calabrese
Last updated by author(s): Jan 26, 2023

Reporting Summary

Nature Portfolio wishes to improve the reproducibility of the work that we publish. This form provides structure for consistency and transparency in reporting. For further information on Nature Portfolio policies, see our Editorial Policies and the Editorial Policy Checklist.

Statistics

For all statistical analyses, confirm that the following items are present in the figure legend, table legend, main text, or Methods section.

n/a	Confirmed	
☐	☒	The exact sample size (*n*) for each experimental group/condition, given as a discrete number and unit of measurement
☐	☒	A statement on whether measurements were taken from distinct samples or whether the same sample was measured repeatedly
☐	☒	The statistical test(s) used AND whether they are one- or two-sided<br>*Only common tests should be described solely by name; describe more complex techniques in the Methods section.*
☒	☐	A description of all covariates tested
☒	☐	A description of any assumptions or corrections, such as tests of normality and adjustment for multiple comparisons
☐	☒	A full description of the statistical parameters including central tendency (e.g. means) or other basic estimates (e.g. regression coefficient) AND variation (e.g. standard deviation) or associated estimates of uncertainty (e.g. confidence intervals)
☐	☒	For null hypothesis testing, the test statistic (e.g. *F*, *t*, *r*) with confidence intervals, effect sizes, degrees of freedom and *P* value noted<br>*Give P values as exact values whenever suitable.*
☒	☐	For Bayesian analysis, information on the choice of priors and Markov chain Monte Carlo settings
☒	☐	For hierarchical and complex designs, identification of the appropriate level for tests and full reporting of outcomes
☒	☐	Estimates of effect sizes (e.g. Cohen's *d*, Pearson's *r*), indicating how they were calculated

Our web collection on statistics for biologists contains articles on many of the points above.

Software and code

Policy information about availability of computer code

Data collection	No Software Used.
Data analysis	ImageJ 1.4.3.67, BiaEvaluation 4.1.1, ASTRA 7.3.1, Coot 8.9.2 EL (ccp4), autoBuster (Global Phasing), ChemLaunch v1.6.8; MassHunter v7.0; BioConfirm v8.0, Pymol 2.4.1

For manuscripts utilizing custom algorithms or software that are central to the research but not yet described in published literature, software must be made available to editors and reviewers. We strongly encourage code deposition in a community repository (e.g. GitHub). See the Nature Portfolio guidelines for submitting code & software for further information.

Data

Policy information about availability of data

All manuscripts must include a data availability statement. This statement should provide the following information, where applicable:

- Accession codes, unique identifiers, or web links for publicly available datasets
- A description of any restrictions on data availability
- For clinical datasets or third party data, please ensure that the statement adheres to our policy

The data that support the findings of this study are available within the main text and its Supplementary Information file. The structural coordinates from X-ray crystallography experiments have been deposited in the RCSB PDB database with the following accession codes: cIAP1Bir3-BCCov (8DSF) and cIAP1Bir3-BCCov-BTKKD (8DSO). Source Data provided as Source Data file. Data is also available from the corresponding author upon request.

Human research participants

Policy information about studies involving human research participants and Sex and Gender in Research.

Reporting on sex and gender	N/A
Population characteristics	N/A
Recruitment	N/A
Ethics oversight	N/A

Note that full information on the approval of the study protocol must also be provided in the manuscript.

Field-specific reporting

Please select the one below that is the best fit for your research. If you are not sure, read the appropriate sections before making your selection.

☒ Life sciences ☐ Behavioural & social sciences ☐ Ecological, evolutionary & environmental sciences

For a reference copy of the document with all sections, see nature.com/documents/nr-reporting-summary-flat.pdf

Life sciences study design

All studies must disclose on these points even when the disclosure is negative.

Sample size	Sample sizes were chosen without calculation. The rationale for assay size and data point numbers was to be consistent with previous publications (Zorba et al PNAS 2018, Schiemer et al NatChemBio 2021)
Data exclusions	No data were excluded.
Replication	In the methods section we've commented on reproducibility of an assay. Briefly, we verified reproducibility in our assays through independent experiments and statistical significance. Ternary complex crystallography was difficult to reproduce, and no pains were taken to identify the rationale for this once a structure was obtained.
Randomization	Randomization was not necessary as we are agnostic to the experimental outcomes, and experimental readouts for BCCov and BCNC are read in parallel with normalization controls.
Blinding	Blinding was not necessary. We have no vested interest in any particular outcome, and the experimental procedures are carried out on model systems or recombinant proteins, rather than "participants."

Reporting for specific materials, systems and methods

We require information from authors about some types of materials, experimental systems and methods used in many studies. Here, indicate whether each material, system or method listed is relevant to your study. If you are not sure if a list item applies to your research, read the appropriate section before selecting a response.

Materials & experimental systems

n/a	Involved in the study	
☐	☒	Antibodies
☐	☒	Eukaryotic cell lines
☒	☐	Palaeontology and archaeology
☒	☐	Animals and other organisms
☒	☐	Clinical data
☒	☐	Dual use research of concern

Methods

n/a	Involved in the study	
☒	☐	ChIP-seq
☒	☐	Flow cytometry
☒	☐	MRI-based neuroimaging

Antibodies

Antibodies used	BTK mAb D3H5 (Cell Signaling Technology Cat. #8547), Vinculin EPR8185 (Abcam Cat. #129002), Anti-rabbit HRP secondary antibody (Life Technologies Cat. #31460), cIAP1 (Clone D5G9 Cell Signaling Technology Cat. #7065), anti-FLAG HRP (Millipore Sigma Cat. # A8592), Streptavidin-HRP (Thermo Scientific Cat. # ENN100), anti-FLAG M2 affinity gel (Millipore-Sigma #A2220).

Validation	D3H5 recognition of BTK was validated by the manufacturer using western blotting against Ramos cells. Vinculin mAb was validated by the manufacturer using western blotting on PC3, HeLa, U937, K562, HUVEC, and HepG2 cell lines. Anti rabbit IgG HRP was validated by the manufacturer using ELISA, western blotting, and co-IP. Anti-Flag HRP was validated by the manufacturer by immunocytochemistry, western blotting, and ELISA. Anti-flag affinity gel was validated by the manufacturer via Co-IP and recombinant protein purification. cIAP1 mAb was validated through western blotting by the manufacturer against THP1, TF-1, KARPAS-299, and 786-0 cells.

Eukaryotic cell lines

Policy information about cell lines and Sex and Gender in Research

Cell line source(s)	SF9 (ATCC CRL-1711), Gibco Expi293 cell line and transfection kit (Cat.# A14524), THP-1 (ATCC-TIB-202)
Authentication	The cells were ordered from manufacturer, but no internal authentication was performed.
Mycoplasma contamination	cell lines were not tested internally.
Commonly misidentified lines (See ICLAC register)	No commonly misidentified lines used here.
